# A traits-based approach to assess aquaculture’s contributions to food, climate change, and biodiversity goals

**DOI:** 10.1038/s44183-024-00065-7

**Published:** 2024-05-31

**Authors:** Aleah Wong, Andrea Y. Frommel, U. Rashid Sumaila, William W. L. Cheung

**Affiliations:** 1 Institute for the Oceans and Fisheries, The University of British Columbia, Vancouver, BC Canada; 2 Faculty of Land and Food Systems, The University of British Columbia, Vancouver, BC Canada

**Keywords:** Marine biology, Biodiversity, Environmental studies, Climate change

## Abstract

Aquaculture has the potential to support a sustainable and equitable food system in line with the United Nations Sustainable Development Goals (SDG) on food security, climate change, and biodiversity (FCB). Biological diversity amongst aquaculture organisms can drive diverse contributions to such goals. Existing studies have assessed the performance of a limited number of taxa in the general context of improving aquaculture production, but few explicitly consider the biological attributes of farmed aquatic taxa at the FCB nexus. Through a systematic literature review, we identify key traits associated with FCB and evaluate the potential of aquaculture to contribute to FCB goals using a fuzzy logic model. The majority of identified traits are associated with food security, and two-thirds of traits linked with food security are also associated with climate change or biodiversity, revealing potential co-benefits of optimizing a single trait. Correlations between FCB indices further suggest that challenges and opportunities in aquaculture are intertwined across FCB goals, but low mean FCB scores suggest that the focus of aquaculture research and development on food production is insufficient to address food security, much less climate or biodiversity issues. As expected, production-maximizing traits (absolute fecundity, the von Bertalanffy growth function coefficient K, macronutrient density, maximum size, and trophic level as a proxy for feed efficiency) highly influence a species’ FCB potential, but so do species preferences for environmental conditions (tolerance to phosphates, nitrates, and pH levels, as well as latitudinal and geographic ranges). Many highly farmed species that are typically associated with food security, especially finfish, score poorly for food, climate, and biodiversity potential. Algae and mollusc species tend to perform well across FCB indices, revealing the importance of non-fish species in achieving FCB goals and potential synergies in integrated multi-trophic aquaculture systems. Overall, this study provides decision-makers with a biologically informed assessment of desirable aquaculture traits and species while illuminating possible strategies to increase support for FCB goals. Our findings can be used as a foundation for studying the socio-economic opportunities and barriers for aquaculture transitions to develop equitable pathways toward FCB-positive aquaculture across nuanced regional contexts.

## Introduction

Aquaculture is expected to play a large role in achieving the United Nations Sustainable Development Goals (SDG), particularly SDG 2 (*End hunger, achieve food security and improved nutrition and promote sustainable agriculture*), SDG 13 (*Take urgent action to combat climate change*) and SDG 14 (*Conserve and sustainably use the oceans, seas and marine resources for sustainable development*)^1^. In this study, based on the UN SDGs 2, 13, and 14 including their targets and indicators, we aim to understand the contribution of aquaculture to food security, climate change and biodiversity goals (FCB).

Increasingly, aquaculture has become an important source of nutrition and livelihoods. Global consumption of aquatic species has nearly doubled since 1961 to 20.2 kg per capita^2^, and fished or farmed seafood provides the primary source of protein for three billion people worldwide^3^. Aquatic foods also contain critical nutrients such as omega-3 fatty acids, iron, zinc, and vitamin A, which could help combat micronutrient deficiencies^4^. The dependence on fish is especially high in rural and low-income communities, particularly in coastal areas or near major river systems^5^. Fisheries and aquaculture together support the lives and livelihoods of around 600 million people, with employment rates growing fastest in aquaculture. In 2020, aquaculture directly employed nearly 21 million people, with many more engaged in processing, catering, and management^2^. While capture fisheries production has remained stagnant after the early 1990s^6^, aquaculture production (excluding algae) reached a record high in 2020, contributing around half of total fisheries and aquaculture production^2^. Aquaculture is believed to increase fish supplies when capture fisheries fail to meet demand^7^ and to bolster the overall resilience of our food system^8^. Here, the assessment of aquaculture’s food security potential is related to the capacity for aquaculture to contribute to SDG 2 with a particular focus on the production of nutritious aquatic foods (Targets 2.1 and 2.2) to eliminate hunger and malnutrition (Indicators 2.1.1, 2.2.1 and 2.2.2). The production potential of aquaculture also depends on food system resilience, sustainability, and productivity (Targets 2.3 and 2.4) and can be indicated by the volume and quality of food production (Indicators 2.2.1, 2.2.2, 2.2.3, 2.3.1).

The growth of aquaculture is uncertain, however, particularly because of the challenges from climate change and aquaculture’s impacts on biodiversity^9^. The growth rate of aquaculture has slowed since its peak in 1996, and production is unlikely to meet projected global demand even with good management^10^. Climate change in particular impacts aquaculture, its dependent sectors, and human communities, posing many challenges for the future development of the sector^11–13^. Climate change is causing ocean warming and acidification, shifts in salinity levels, increasing intensity and duration of droughts and freshwater shortages, increasing eutrophication and disease episodes, and other extreme weather events^14–16^. These climate-induced environmental changes are impacting aquaculture productivity directly by changing the ecological capacity for aquaculture^13^ and indirectly through infrastructure damage and higher feed costs due to declining crop yields and forage fish landings^9,17^. Freshwater aquaculture production quantity as well as food safety are greatly affected in most countries, as a result of warming, changes in precipitation, and eutrophication. In developing countries, anthropogenic impacts on freshwater aquaculture are high, and national response capacity is low^18^.

Climate change impacts on aquaculture will affect food security and livelihoods since aquaculture production is an important source of protein and nutrients as well as a major source of income through trade and exports^19^. Communities with existing food access challenges, particularly those who depend on aquaculture at the subsistence level, will be particularly vulnerable to climate change-related decreases in production^20^. In this study, we use “climate change potential” to refer to the extent of aquaculture’s contributions to SDG 13, particularly through production approaches that “strengthen resilience and adaptive capacity to climate-related hazards and natural disasters” (Target 13.1, Indicator 13.1.3) and the explicit consideration of climate change in national policies and strategies (Target 13.2, Indicators 13.2.1 and 13.2.2) concerning aquaculture.

Meanwhile, aquaculture also impacts biodiversity and its environmental footprint^9^. High freshwater demand and the conversion of sensitive areas like mangroves and wetlands into aquaculture systems reduce native habitat and biodiversity. Escaped individuals, diseases, effluents, and chemicals from aquaculture operations drive changes in environmental quality and communities, with cascading effects on ecosystems^21–24^. Aquaculture also intensifies overfishing due to reliance on wild fish stocks for feed^25^. According to life cycle assessments, feed is responsible for over 90% of the environmental impact of fed aquaculture^26,27^, and despite some improvements in feed efficiency, many farmed species like salmon still consume more food than they produce^28^. The use of wild-caught fish feeds in aquaculture has implications for local food security, since some households rely on small pelagic fish that are becoming less and less available as a result of the fishmeal industry^29–31^. The United Nations Sustainable Development Goal 14 highlights biodiversity targets to reduce marine pollution (Target 14.1, indicator 14.1.1), use ecosystem-based approaches to sustainably manage marine and coastal ecosystems (Target 14.2, indicator 14.2.1), and end overfishing to restore healthy stocks (Target 14.4, indicator 14.4.1). In order to maximize aquaculture’s ability to feed our growing population, we need strategies to increase the resilience of aquaculture and food security to global change while mitigating or reversing its negative impacts on climate change and biodiversity^8^.

## Traits-based approaches

The traits of farmed species can influence sustainable aquaculture development at the food security, climate change, and biodiversity conservation nexus (FCB) because biological and ecological characteristics predispose certain species for successful domestication and production^32,33^. Studies have recognized that biological and ecological traits influence species distributions^34^ and can be used as indicators of invasiveness^35^, sensitivity to fishing^36^, and climate change^37^. Traits such as growth rate, aggression, and tolerance to rearing density have even been used to evaluate the suitability of species for aquaculture^33,38,39^. Characterization of traits of farmed species and strains has been a major subject in aquaculture, particularly for selective breeding and genetic modification to increase the species’ suitability for specific aquaculture objectives^40^. Trait-based analyses can thus provide biological and ecological context to evaluate the performance of individual species, making this framework particularly suitable to evaluate the potential of aquaculture species to contribute to food security, climate change, and biodiversity goals.

While previous research has identified ecosystem-level effects of aquaculture on biodiversity and global-scale climate change impacts on aquaculture, most trait-based analyses concern a single taxa or single trait at once with the objective of maximizing aquaculture production and are used to evaluate new species for production rather than to assess current species^32^. Single-trait assessments, which are often solely focused on the growth rate of aquaculture species^41,42^, can neglect key differences between populations and produce incongruous results between different traits studied. Multi-trait approaches can consider a set of features, but are more time-consuming, expensive, and may reflect only one biological function such as growth or production quality^32,43^.

In this study, we develop a traits-based approach to assess the suitability of farmed species in contributing to FCB objectives. Through a systematic literature review, we identify traits of farmed aquaculture species that are related to FCB objectives. We then adapt a fuzzy logic system framework to quantify the potential of aquaculture species to contribute to FCB objectives. Such a fuzzy logic framework has previously been applied to study marine species’ traits in relation to risks and vulnerability of marine fishes to fishing and climate change^36,37^. Fuzzy set theory, or “fuzzy logic”^44^ is a robust approach to analyzing data with gaps and uncertainty because it allows subjects to be classified into multiple categories at varying levels of membership, and can be updated when data and understanding are improved. This framework can accommodate the lack of efficient trait measurement methods and data availability, consider within-species trait variability^45^, and account for uncertainty in our conclusions to better inform decisions when selecting candidate species for aquaculture. We designed this fuzzy system for a global quantitative analysis to identify species with the greatest potential to support FCB goals and reveal priorities for transitions in aquaculture, by incorporating traits that impact FCB. The framework developed in this study is intended to inform strategies to meet the UN’s SDG goals and illuminate potential opportunities for livelihood transitions in aquaculture.

## Results

### Most aquaculture traits are associated with food

Sixty percent of aquaculture traits identified during the systematic review are associated with food security. Many traits overlap across FCB categories, though overlap is most common with traits associated with food security. Two-thirds of the traits associated with food security are also associated with either climate change or biodiversity. By contrast, around two-fifths of traits and one-third of traits are associated with climate change and biodiversity, respectively, and less than half of the traits associated with either climate change or biodiversity are associated with another FCB dimension.

### Most species have intermediate FCB scores

We find narrow to intermediate ranges in the FCB index values when considering the absolute scores of species (from 1-100). Food security scores span a range of around 30 index points, while climate change and biodiversity scores span a range of around 40 index points. The mean food security, climate change, and biodiversity scores across all 54 analyzed species are 50, 46 and 53, respectively.

### Taxonomic differences in FCB potential

The parametric food, climate, and biodiversity scores reveal significant differences in FCB potential between taxonomic groups, which makes sense because most of the identified traits used in our analysis are ubiquitous to taxonomy. The scores were non-normally distributed and thus we chose to compare the results using Kruskal-Wallis tests (SI 1.4, Tables S15-S17.)

**Fig. 1:**
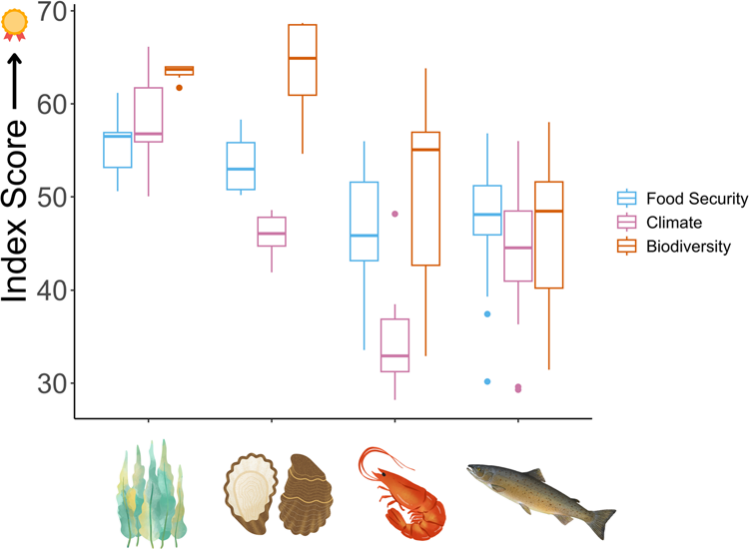
Food security, climate change, and biodiversity scores by taxonomic group. The bottom and top of the boxplots show the 25th and 75th quartiles of the FCB scores, respectively. The lower and upper end of each vertical line represent the minimum and maximum values, while the dots are outliers. The line in the center of each boxplot represents the median FCB score for each taxonomic group.

As expected, non-fed species tend to score better for FCB than fed species at the taxa level. According to post-hoc Dunn’s tests, algae and molluscs have significantly different (higher) median food and biodiversity scores from finfish and crustaceans (Fig. 1). Algae and molluscs do not have significantly different median scores for food security or biodiversity scores, but algae species have higher median scores for climate potential than molluscs. Crustaceans have significantly lower climate scores compared to all other taxonomic groups, while molluscs and finfish do not differ significantly in median climate scores. Finfish and crustaceans do not have significantly different median scores for food security or biodiversity scores, although scores within taxa vary greatly for these taxa, especially biodiversity scores. This is likely in part due to the wide variety within these taxonomic groups--especially the finfish group, which includes species with trophic levels between 2 and 5, for example. Indeed, Table 1 reveals how lower trophic finfish and crustaceans tend to have higher raw FCB scores.

**Table 1:**
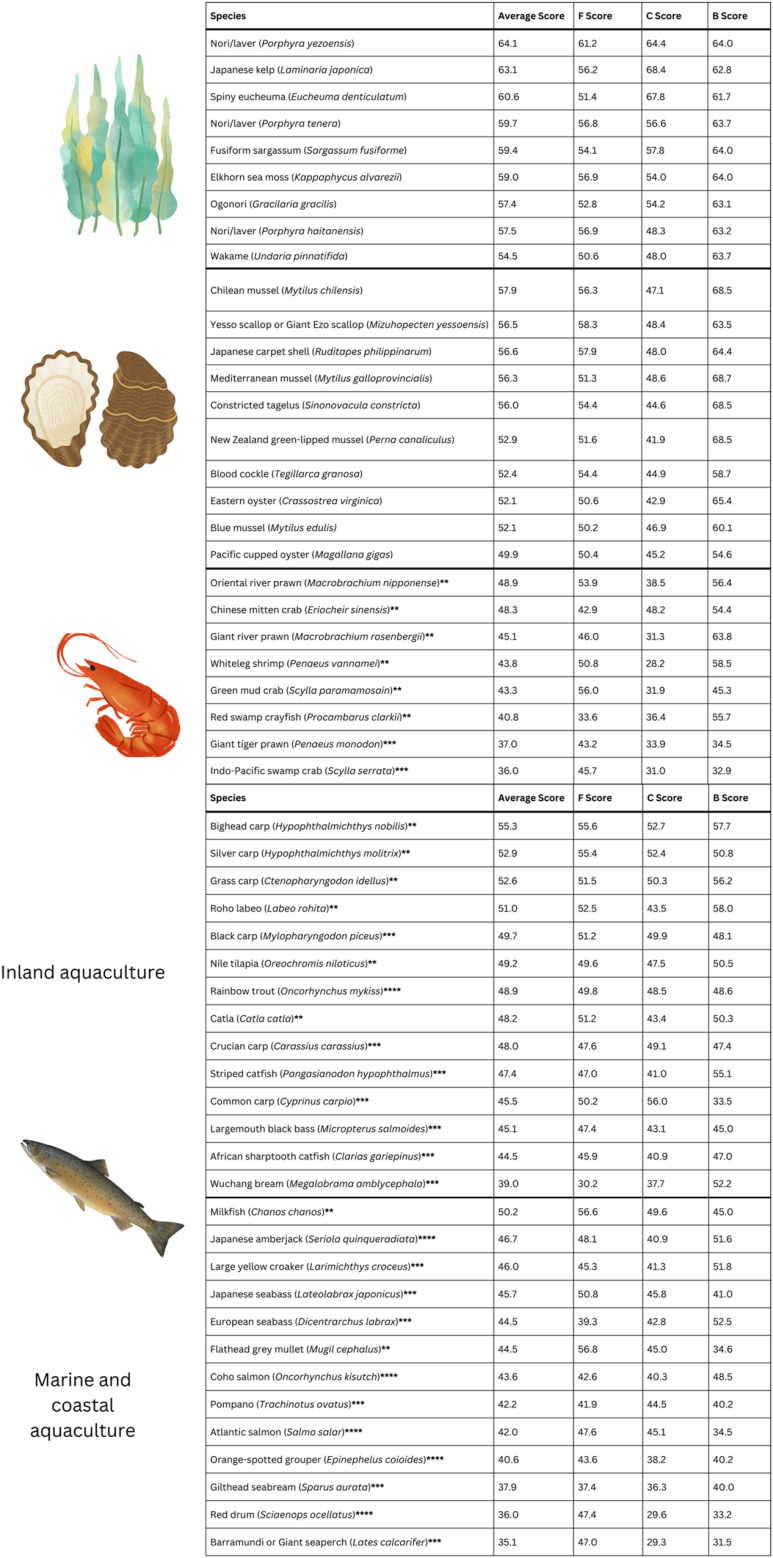
Species scores for food security, climate change and biodiversity potential, organized by taxonomic category

The higher the score, the greater the potential of the species to contribute to the FCB goal. “Average score” is the mean score for each species across each FCB category. Species within each taxonomic grouping are listed from highest to lowest average score. Asterisks by finfish and crustacean species names denote trophic level (** denotes TL < 3; *** denotes TL < 4; **** denotes TL ≥ 4). See Table S14 for non-parametric rankings, from the best- (#1) to worst-scoring (#54) species in each FCB category.

Our calculations for individual species show that some species of finfish and crustaceans score relatively high for food, climate, and/or biodiversity based on their unique traits. This level of nuance for major aquaculture species obtained from our trait-based approach differs from traditional assessments which typically target only a few species at a time on the basis of a couple traits. Species-level information about FCB potential derived from inherent, biological characteristics may inform targeted, effective strategies to improve the FCB potential of aquaculture beyond simply switching one aquaculture species for another (e.g., selection of a portfolio of complementary species to achieve FCB targets and alleviate trade-offs).

Raw scores calculated by the fuzzy system reveal species-specific differences and tradeoffs in FCB potential, but differences between species are sometimes represented by a difference of several index points (or less). Since the range of F, C, and B scores occur within 30 and 40 index points, one index point could indicate a notable difference in FCB potential. Thus, we have chosen to include non-parametric rankings for species (#1-54), as this can provide more statistical power because FCB scores are not normally distributed. Non-parametric rankings of species’ FCB potential could be used as a tool for policymakers to compare species broadly, both within and across taxa. We ranked species based on their potential in each FCB category as well as an overall rank based on the mean score in each category (Table S14).

The mean scores of the ten highest-ranked species in each FCB category (Table S14) are significantly different from the mean FCB scores of all analyzed species (Kendall rank test; *p* = 9.83 e−11 for F; *p* = 2.20 e-06 for C; *p* = 4.40 e−05 for B). Similarly, the mean scores of the ten lowest-ranked species in each FCB category are significantly different from the mean FCB scores of all analyzed species (*p* = 5.00 e−05 for F; *p* = 0.004 for C; *p* = 0.04 for B).

The ten species with the lowest potential to contribute to food security, climate change, and biodiversity goals belong to crustacean and finfish taxonomic groups (Table 1, Table S14). Gilthead seabream (*Sparus aurata*) and giant tiger prawn (*Penaeus monodon*) are amongst the lowest ten species for every FCB category. However, two species of finfish, flathead gray mullet (*Mugil cephalus*) and milkfish (*Chanos chanos*), are in the top ten species for food security, and three finfish are in the top ten for climate change potential: common carp (*Cyprinus carpio*), bighead carp (*Hypophthalmichthys nobilis*) and silver carp (*Hypophthalmichthys molitrix*). However, flathead gray mullet and common carp score in the bottom ten for biodiversity potential. The only crustacean species in the top ten for biodiversity potential, the giant river prawn (*Macrobrachium rosenbergii*), scores in the bottom ten for climate change potential.

The top ten species for biodiversity potential tend to be molluscs and algae. Every algae species in the top ten for food security is also in the top ten species for biodiversity potential. Algae comprise the majority of species with high potential to contribute towards climate change goals, along with three species of finfish. Every algae species in the top ten for biodiversity potential is also represented in the top ten species for climate change potential. Furthermore, the algae species laver/nori (*Porphyra yezoensis* and *Porphyra tenera*), elkhorn sea moss (*Kappaphycus alvarezii*), and Japanese kelp appear in the top ten species for every FCB category. Across all three FCB categories, the species with the most potential are algae and molluscs.

### Comparison of Atlantic salmon, Japanese kelp, and Pacific oyster

Compared to Atlantic salmon (*S. salar*) and Pacific oyster (*M. gigas*), Japanese kelp (*S. japonica*) has the highest potential for every index (food security, climate change, and biodiversity indices). The high FCB potential of *S. japonica* could indicate that a similar, native species, sugar kelp (*S. latissima*), may also have great potential to contribute to food, climate, and biodiversity goals in its native habitat, including British Columbia. Pacific oyster and Atlantic salmon have similar scores for climate change, but Pacific oysters scores higher for food security and biodiversity. Out of 54 analyzed species, Japanese kelp has the greatest potential to support climate change goals, while Atlantic salmon is second-to-last in terms of biodiversity potential. However, neither Atlantic salmon nor Pacific oyster are native to British Columbia, and both species have had deleterious effects on native species^46,47^. Farmed outside their native ranges, algae species can also become invasive, as evidenced by the introduction and rapid spread of Japanese kelp in China^48^. The ecological and socio-ecological consequences of Japanese kelp outside its native range remain unclear, but the potential impacts of invasive algal species are well-documented^49^. These impacts include introducing parasites and pathogens to a new environment, genetic changes in native populations as a result of hybridization, altered physico-chemical conditions, nutrient cycling, and energy flows^50^.

When compared to the mean FCB profile, Atlantic salmon and Pacific oyster have relatively average food security and climate change scores, but Atlantic salmon has a below-average biodiversity score and Pacific oyster scores around the average biodiversity score. In contrast, Japanese kelp has above-average scores for all FCB indices and has particularly strong potential for climate change and biodiversity goals. According to non-parametric rankings, Japanese kelp ranks in the top half (1–27) of species for all three indices, while Pacific oyster and Atlantic salmon rank in the bottom half (28–54) for food security, climate change, and biodiversity indices, although FCB rankings for Pacific oyster are around the median rank of 27.

### Correlations between food security, climate change and biodiversity

We expected some covariation between food security, climate change, and biodiversity indices because the fuzzy system was designed with some traits that influence multiple indices. Traits related to multiple indices had a larger influence on species’ averaged performance scores, which could be useful for holistic assessments of species across FCB categories because trait overlap and correlations across FCB categories demonstrate the inherent connectivity of these three issues. Moderate covariation reflects the assumptions in our system, and we are able to account for uncertainty about the extent to which traits contribute to different goals by using fuzzy logic. Using our methods, alternative assumptions could be tested about how traits are linked with food, climate, and biodiversity, and how different species or individual traits could contribute to FCB objectives.

Since the food security and biodiversity scores were not normally distributed according to the Shapiro-Wilk normality test and upon visual inspection, we used the Kendall rank correlation coefficient (Kendall’s tau statistic) to estimate rank-based measures of association between food security, climate change, and biodiversity scores. Kendall correlations are preferred for small sample sizes, which is appropriate for our analysis of 54 species^51^. Food security and climate change shared six traits in common and were positively correlated with a tau value of 0.441 (*p* = 1.299e−06). Three traits were associated with both food security and biodiversity, which were positively correlated with tau = 0.377 (*p* = 3.608e−05). Climate change and biodiversity were correlated to a lesser degree with a tau of 0.283 (*p* = 0.00195), which makes sense because C and B shared only one trait in common.

### Sensitivity analysis

**Fig. 2:**
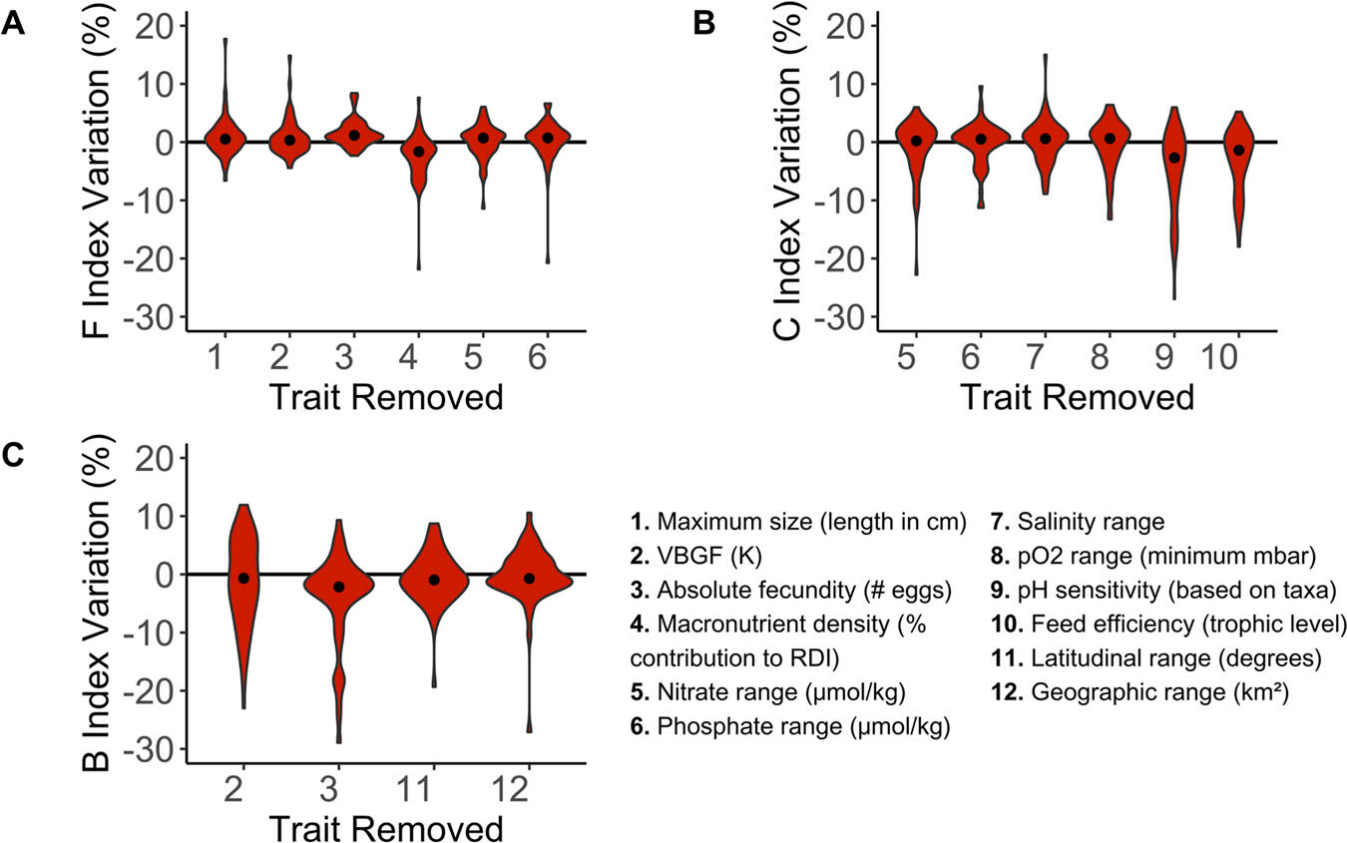
Jackknife analysis of food, climate, and biodiversity indices. Percent variation in species’ index values of food security (**A**), climate change (**B**), and biodiversity (**C**) following removal of traits as inputs to the fuzzy system. Percent variation was calculated as the deviation of index points from the baseline (index when all traits were included) divided by the maximum index score. The maximum scores for food security, climate change, and biodiversity, respectively, were 61, 66, and 68 index points. Figure 2 shows only traits that result in a variation of 10% or more when removed from the system. The black dots are the median of the percent variation of the 54 aquaculture species when attributes were removed. Violin plots show the minimum, maximum, and distribution of deviations. See SI 5 and Figure S3 for more details about the sensitivity analysis.

Jackknife analysis showed that for the majority of species, FCB scores varied within a small range equal to 5% of the maximum food, climate, or biodiversity score (Fig. 2). For some species however, maximum deviations reached 20–30% the maximum FCB score in either the positive or negative direction.

The food security index was sensitive to maximum size, the von Bertalanffy growth function coefficient K (hereafter VBGF K), absolute fecundity, macronutrients, nitrate, and phosphate range (Fig. 2A). Phosphate and nitrate ranges were included as traits in the food security fuzzy system because nutrients are common in high-density rearing environments and tolerance to high nutrient levels can be beneficial for production^52^. When maximum size, the VBGF (K), and nitrate range were removed from calculations, food security indices varied by over 10% of the maximum food security score, 61. When absolute fecundity, macronutrients, and phosphate range were removed from calculations, food security indices varied by around 20% of the maximum food security score. Removal of macronutrient density, nitrate, and phosphate ranges tended to result in unsymmetrical negative bias on the predicted food security index. In contrast, excluding maximum size, VGBF (K), and absolute fecundity tended to result in positive bias.

The climate change index was most sensitive to nitrate range, phosphate range, salinity range, pO2 range, sensitivity to pH, and feed efficiency (using trophic level as a proxy) (Fig. 2B). Climate index scores varied by over 10% of the maximum score (66) when phosphate range, salinity, pO2 range, and feed efficiency were removed from calculations. When nitrate range was removed from calculations, climate indices varied by over 20% of the maximum climate index. When pH sensitivity was removed from calculations, climate indices varied by over 30% of the maximum climate index. The exclusion of nitrate range, phosphate range, pO2 range, pH sensitivity, and feed efficiency from the system resulted in negative asymmetrical biases on the climate change index. Removing the salinity range resulted in a positive asymmetrical bias. The biodiversity index was most sensitive to VBGF (K), absolute fecundity, latitudinal range, and geographic range (Fig. 2C). Removal of each of these traits resulted in unsymmetrical negative bias on the biodiversity index, with variation in index scores exceeding 25% of the maximum biodiversity index score (68) for every trait listed above except latitudinal range.

## Discussion

### Limitations of aquaculture’s focus on macronutrients

The vast majority of traits identified in the systematic review were associated with food security through their connection to aquaculture productivity. However, while our fuzzy system placed most species in the “Very high” fuzzy set for macronutrients, the final food security indices across 54 species span only a narrow range of scores, and species typically associated with food security score lower than expected. Although salmonids and other small pelagic fish have been reported as the most nutrient-dense seafoods^53^, we find that the highest-ranking species on a more holistic index of food security include molluscs and algae. This is likely due in part to the rich micronutrient content of algae such as omega-3 and omega-6 polyunsaturated fatty acids, vitamins A, C, E, B12, and iodine^54^. The importance of micronutrients to nutrient density scores has been documented, especially niacin, vitamin D, and vitamin B12, which had the greatest impact on nutrient density in Bianchi et al. ^53^.

Low mean FCB scores may reflect the shortcomings of aquaculture’s focus on the production and nutrients of aquatic species. This singular focus may overshadow important considerations about FCB trade-offs as well as the social-economic factors affecting food security. For example, high-yield production and economic value are not necessarily conducive to local food security^55^. Most farmed fish are exported rather than used for food at the subsistence level^56^ and species with low market value end up feeding local people, crucial for food security and rural development^30^. Studies of agendas to promote aquaculture have found that while production increased, income and food access stayed stagnant for low-income households. For other communities, the emphasis on increasing aquaculture output has transformed historically subsistence fisheries into commercial systems producing wealthier urban markets^57,58^.

Our results show that traits associated with food security tend to be associated also with climate and biodiversity. Thus, focusing primarily on traits beneficial for food production may ignore important FCB trade-offs. For instance, species with large natural distributions, tolerance to a wide range of environmental conditions, high fecundity, and fast growth rates tend to be successful invaders because they can outcompete native species^59^. These same traits also allow for resilient and efficient production. In our analysis, species with extreme values for absolute fecundity and VBGF (K) were placed in both “Low” and “Very high” fuzzy sets for their potential to contribute to biodiversity and food security, respectively, because these traits have opposite conclusions for those indices. Because absolute fecundity and VBGF (K) were highly influential on both food security and biodiversity indices but in opposite directions, moderate values for these traits may be favorable in mariculture, while higher values may be acceptable for contained aquaculture systems with minimal risk of escapes. Trade-offs between food security and the other two indices are common because production focus tends to be economic and related to food rather than environmental considerations^32^. For instance, the intensive farming of non-native Atlantic salmon in British Columbia has significant economic value but releases invasive individuals, waste, pathogens, and parasites that threaten wild species^60–62^.

### Changes to aquaculture production, with special attention to trophic level and environmental range traits, could improve FCB potential

Traits related to trophic level and environmental range influence multiple FCB categories and have the potential for co-benefits, synergies, or trade-offs in FCB impact. While inherent, unmodified traits can estimate species performance across contexts, this study provides additional information to identify traits (or the effects of traits on FCB) that can be manipulated to improve FCB potential through targeted breeding programs. Our results also reveal where advances in production are important to improve FCB potential. For example, special consideration should be taken to ensure species with low biodiversity potential are farmed in their native areas or in land-based systems outside their natural geographic range. Climate discussions are beginning to pay greater attention to the environmental impacts of food systems, and policymakers need accessible, holistic information on aquaculture species in order to develop measures that support efforts to mitigate and adapt to climate change (such as Nationally Determined Contributions under the Paris Agreement). FCB scores calculated from biological traits can help inform scalable, species-specific, or trait-focused strategies to increase FCB potential and aid decision-makers in evaluating the risks and potential trade-offs of such interventions.

Despite data gaps for non-fish species, low-trophic algae, and mollusc species tended to score highly in multiple FCB categories, revealing the synergistic nature of these taxonomic groups. Some algae species like *P. yezoensis* and *P. tenera* (both commonly known as laver/nori), elkhorn sea moss, and Japanese kelp have a high potential for food security, climate change, and biodiversity, so farming these species could contribute simultaneously to FCB goals. Our observations align with current understanding of the social and ecological impacts of farming species at higher trophic levels. Farming high trophic-level species directly threatens food security in subsistence fishing communities by depleting small pelagic fishes for use in aquafeeds^30,31^. Dependence on feed-inefficient aquaculture can also indirectly affect food security, since these livelihoods and income are subject to the economic volatility of fluctuating forage fish stocks^63^. Moreover, aquaculture of high trophic species strains marine biodiversity through overfishing and bycatch associated with acquiring feed, also requiring greater energy usage^25^.

Although low trophic level mollusc and algae species tend to have higher FCB potential relative to higher-trophic species, traits other than trophic level were influential on FCB scores and could be targeted to maximize non-fish food security potential. While the degree to which the nutritional content of a species can be influenced (e.g. through the timing of harvest and feed composition) is limited^53^, food security potential of molluscs and algae can be improved with the development of breeding technologies to enhance nutrition and productivity, by changing cultivation practices^64^, increasing aquaculture leases by streamlining the leasing process^65^, and cultural shifts that encourage consumption of seaweeds and molluscs (e.g. fostering producer-consumer relationships and enhancing community access to locally-grown products)^66^. Ocean-based climate solutions such as carbon capture and ocean-based renewable energy are only just beginning to attract attention^67,68^, but little is known about the potential for these solutions to simultaneously support food security.

Food and biodiversity co-benefits likely exist in mollusc aquaculture, with species that perform best on the food security index also scoring well on the biodiversity index (e.g. Japanese carpet shell, *R. philippinarum* and Chilean mussel, *M. chilensis*). Given that many seaweeds are also recognized as a low-impact food source^69^, combining the culture of seaweeds and molluscs in regenerative ocean farming could help increase production while promoting ecosystem recovery^70^. Studies have shown that filter-feeding molluscs and algae can reduce eutrophication^9,71^ and potentially offset greenhouse gas emissions by sequestering carbon in shells and through photosynthesis^72–74^. Of course, any benefits to biodiversity will be determined in large part by the species’ invasiveness, which can depend on the location and production type (open versus closed system). Integrated multi-trophic aquaculture (IMTA) could further harness synergies by farming bivalves, seaweeds, and finfish together, thereby utilizing waste from higher-trophic species as fertilizer for lower-trophic species and mitigating the environmental impacts of excess nutrients^64,75^. Cao et al. ^18^ have shown that seaweed mariculture is the least vulnerable form of blue food production to anthropogenic change, suggesting its potential for climate adaptation and mitigation. IMTA could provide a way for aquaculture farmers to increase the FCB potential of their production while diversifying their income and building resilience to environmental or economic shocks^8^.

Particular attention should be given to improving the FCB potential of crustacean and finfish species that scored poorly for multiple indices such as giant tiger prawn, gilt-head bream, and Wuchang bream, and species with large FCB trade-offs. For instance, flathead gray mullet and green mud crab have a high propensity to contribute to food security, so it may be worth investigating ways to reduce their impacts on climate change and biodiversity. These disparate food, climate, and biodiversity scores reflect the complexity of species selection and point to the need for targeted interventions to balance FCB trade-offs. Selecting species to farm based on their inherent, biological potential to support FCB goals is a useful starting point to improve aquaculture outcomes because of the risks and high cost of genetic modification and developing new production technology^40^ and is already used widely to capitalize on inherent, desirable traits such as growth rate^32,76^. Species selection is considered an effective short-term intervention to increase the environmental performance of aquaculture and could be particularly relevant to the development or expansion of aquaculture if consideration is given to how species’ traits interact with different production systems. Given that only a small percentage of aquaculture production is based on genetically improved stocks^77^, and that production technologies that could reduce negative biodiversity impacts of aquaculture are still emerging, there is great potential for species-specific and trait-specific information to help tailor efforts to improve aquaculture’s FCB potential.

Policymakers can encourage the farming of FCB-positive species by redirecting fiscal policy away from low-scoring species and harmful practices towards sustainable production, which could include co-culture systems. Investment in species diversification and breeding programs could help aquaculture harness benefits and synergies of species with untapped potential to contribute to FCB goals, while funding for research and development of innovations like novel plant-based feeds could enable more sustainable production of economically important species, especially by reducing the environmental impacts of acquiring wild-caught fishmeal^78,79^. Of course, production shifts and genetic modification come with their own constraints and risks^64,80^. Any intervention is likely to come with its own suite of FCB trade-offs. Our approach, however, aids in identifying priority species and FCB disparities where such interventions (and their own trade-offs) should be explored.

### Socio-ecological influences on FCB potential

We estimate biological and ecological FCB potential using the assumptions and inputs of the fuzzy system. Our calculated scores represent the relative FCB potential of species across contexts with and without considerations in place to improve FCB. While we do not explicitly account for extrinsic factors such as production technology in species’ FCB scores, our results help identify species with unmet potential and the socio-economic barriers to achieving a species’ full potential. According to our particular definition of food security which is based on the specific set of attributes identified by the literature, some algae species have high food security potential. Though seaweeds are not currently known to contribute substantially to macronutrient requirements^81^, and only around 31% to 38% of farmed seaweed is currently consumed directly as food^63^, algae species score highly for food security potential due to their micronutrient content and potential climate resilience. Here, we distinguish between FCB potential and whether a species’ potential is utilized. For instance, elkhorn sea moss (*K. alvarezii*) is currently grown primarily for carrageenan, a stabilizer, and emulsifier^82^, but its high FCB potential suggests that shifting seaweed production and use to direct human nutrition will likely have the most influence on food security. Similarly, we estimate that sugar kelp will have great FCB potential like its close relative Japanese kelp, when farmed in its native range. Since sugar kelp aquaculture is still developing in North America, and grown for a variety of purposes beyond human consumption, the species’ potential is yet unrealized.

The economic structure of global aquaculture may also pose challenges to harnessing FCB potential. In the case of algae, the vast majority of global seaweed production is concentrated in Asia^83^ and there is an oversupply of Wakame (*Undaria pinnatifida*) in Korea and Japan^84^. Without a large market for seaweeds in North America, starting new farms could be a financial risk. In fact, in the 1980s, *Pyropia* farming failed to gain footing in Washington state due to leasing capacity issues^85^. The nascent seaweed industry in British Columbia faces similar challenges with uncertainties and delays in obtaining leases and the lack of a seaweed-specific regulatory framework^65^. These challenges have historically led to reluctant or unwilling investors^86^ and will likely limit the extent to which seaweed aquaculture can grow and contribute to FCB goals. Because the seaweed sector in North America is still nascent, research should be conducted to evaluate the economic and ecological limits of seaweed species’ food, climate, and biodiversity potential.

Despite the limitations described above, our generalized approach is a strength of this study because it enables global analysis of a wide range of taxa, including species with limited information. Our trait-based approach is particularly helpful in this study because it facilitates the integration of a broad range of biological and ecological knowledge to simplify the complexities of intertwined issues such as FCB. Trait-based approaches also offer predictive power and are efficient and scalable^87^. Combined with our use of fuzzy logic, we are able to overcome the methodological limitations of the multi-trait approach, including the lack of efficient methods to measure some traits and the risk of error when deriving conclusions from multiple traits^32^. With increasing resources, technology, and data, our fuzzy expert system can be updated to reflect the most current understanding of relationships between FCB and species and more changes can be made to improve the FCB potential of aquaculture production^88^.

### Future directions for research

Future applications of a trait-based analysis could help decision-makers prioritize aquaculture improvements depending on local socio-economic conditions. For instance, our broad taxonomic comparison helps identify context-specific gaps and priorities regarding aquaculture shifts in British Columbia, where Fisheries and Oceans Canada (DFO) is re-strategizing the future of salmon farming^89^ and seaweed aquaculture is emerging as a potential environmental solution^90^. As two of the most farmed species in B.C., the FCB profiles of Atlantic salmon and Pacific oyster provide insight into provincial aquaculture’s support for food security, climate change, and biodiversity goals. To improve the FCB potential of aquaculture in British Columbia, actions could be dedicated to increase the biodiversity potential of Atlantic salmon. Land-based systems, or culturing Atlantic salmon with filter-feeding bivalves like Pacific oyster and with seaweeds like sugar kelp in an integrated multi-trophic aquaculture system (IMTA) could mitigate some of the negative biodiversity impacts of farming^64,75,91^. IMTA systems combining Atlantic salmon, blue mussel (*Mytilus edulis*), and sugar kelp are currently operating on the Northeast coast of Canada, and they have been found to be more profitable and economically resilient than salmon monocultures^92^. The variety of blue mussel most commonly farmed in B.C. (*M. edulis*) is non-native and is known to outcompete local mussel varieties^93^. As such, future research on multi-trophic aquaculture should target the performance and potential benefits of native blue mussel (e.g., *M. trossulus* in B.C.) in IMTA systems as well as on the socioeconomic implications and feasibility of livelihood transitions in seaweed aquaculture^94^.

Furthermore, we recognize that food security is influenced by many complex and context-dependent factors which can be explored in additional analyses to augment our use of food production-related traits. Because abundance is strongly linked to availability, and increasing production is expected to improve both affordability and consumption in all countries^95^, our calculation of the F index reflects the importance of food production to food security, while highlighting the interconnectedness of food production with climate and biodiversity. For example, maintaining or increasing production under climate change has implications for food prices, global trade, and consumption. Species’ F indices could help national policymakers formulate guidelines for in-country aquaculture production, consumption, and imports to improve local food security based on country-specific information on existing infrastructure, diet preferences, and environmental constraints for aquaculture.

Future studies can employ a social-ecological systems (SES) approach to ensure the biological potential of aquaculture matches with existing social needs and assets (e.g., licensing infrastructure, regulations, market, training programs, etc.)^96^. Developing a framework to incorporate social-ecological traits into the evaluation of aquaculture’s FCB potential should be a priority, as it is currently lacking. Social-ecological traits-based approaches have great potential to help researchers, policymakers and stakeholders better navigate complexities at the intersection of food, climate, and biodiversity^97^. For example, social-ecological traits could encompass additional dimensions of food security including food distribution and access. Such an approach will build upon the biological underpinnings of aquaculture production opportunities and limitations, and further enable the analysis of aquaculture production across diverse and complex contexts.

Building off of our approach, studies could use species trait data across different environmental conditions and production systems (e.g., intensive vs. extensive) to examine FCB potential both inside and outside a species’ natural range. This analysis can be applied to a variety of production contexts across geographic regions to generate a nuanced understanding of variations in FCB potential within a single species, especially farmed species like carp and tilapia which are farmed in a wide range of contexts. Further research will need to consider the biological potential of species alongside social-ecological dimensions of FCB including decision-making in aquaculture, livelihood transitions, and globalized markets^98,99^. Importantly, shifts in aquaculture to work towards FCB goals necessitate the consideration of socio-economic conditions needed to accommodate FCB trade-offs, including tangible transition pathways for communities and individuals involved in aquaculture supply chains. The emerging North American seaweed sector and IMTA systems in particular could use research attention, as sector growth is constrained by knowledge gaps on species potential to support ocean-based solutions^72^ as well as on the long-term outlook and sustainability of livelihoods^65^.

This study illuminates future priorities and potential opportunities to help aquaculture meet the UN’s Sustainable Development Goals on food security (SDG 2), climate change (SDG 13), and biodiversity (SDG 14). Given the global scope and intertwined nature of food security, climate change, and biodiversity issues, particularly in the aquaculture sector, it is crucial to have a biologically-informed approach to assess the FCB potential of a broad range of aquaculture species. Using a traits-based approach is data-efficient, accounts for uncertainty, and can help inform decisions during aquaculture production development and management to support FCB goals. Our research suggests that we can improve the FCB potential of aquaculture using strategies that consider specific species’ food-climate-biodiversity trade-offs. Ultimately, we identify a need to expand the focus of aquaculture research and development to include the nexus of food security, climate change, and biodiversity.

## Methods

### Systematic literature review

**Fig. 3:**
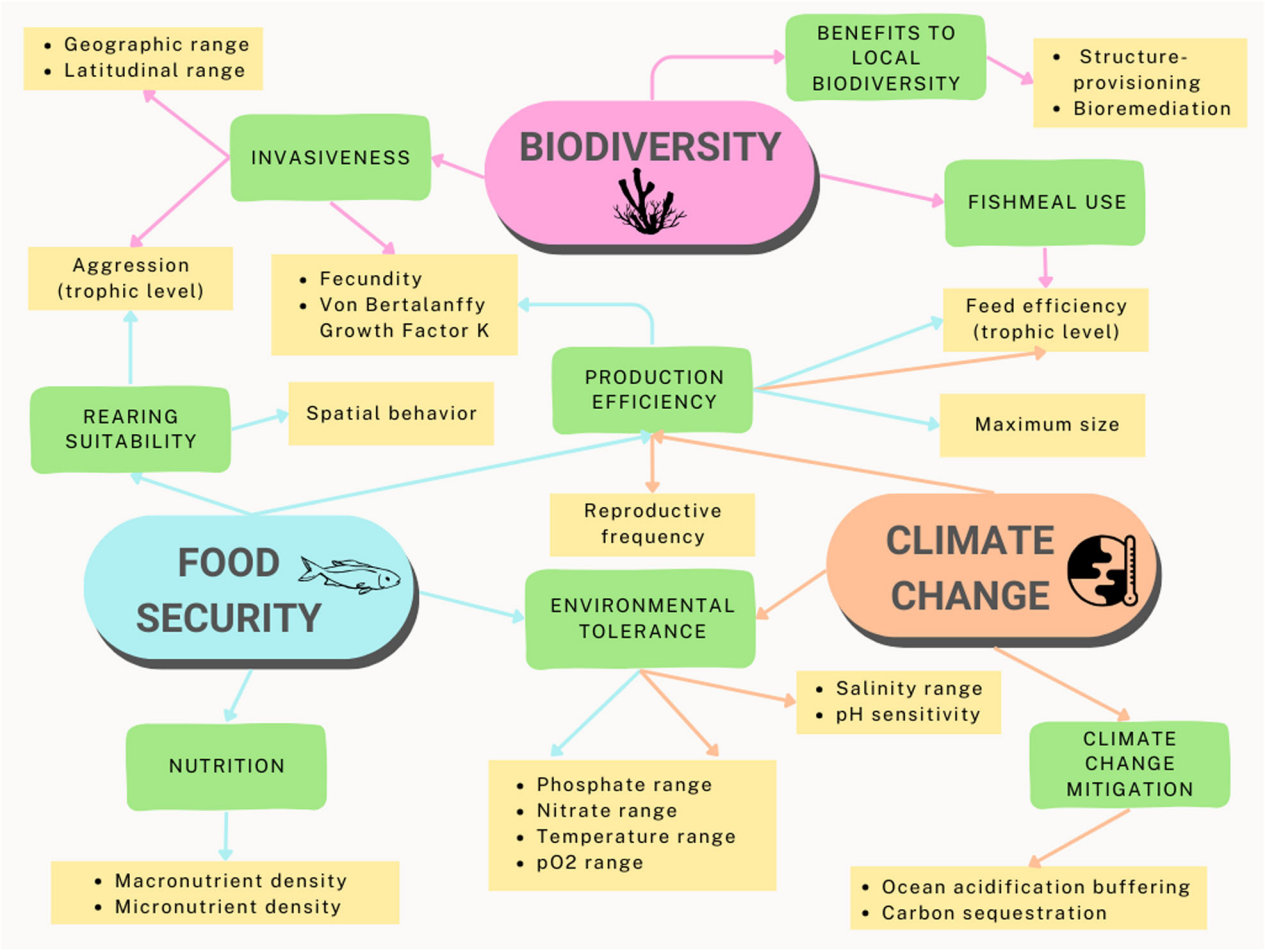
FCB themes and traits synthesized from the systematic literature review. Themes corresponding with each of the three FCB dimensions are capitalized in green boxes. Blue, orange and pink lines connect food security, climate change and biodiversity (respectively) with their associated themes and traits. Traits corresponding with each theme appear in yellow boxes.

To obtain a list of traits (Fig. 3) of farmed aquatic species that are associated with food security, climate change, and biodiversity, we conducted a qualitative systematic review of the peer-reviewed literature based on the protocol outlined in the PRISMA (Preferred Reporting Items for Systematic Reviews and Meta-analyses) statement^100^ (See SI 1.2). We targeted publications available on Google Scholar that discussed biological or ecological characteristics of farmed aquatic species in relation to FCB and recorded the following data from each study: (1) the trait mentioned, (2) the FCB context surrounding the trait: F, C, B, or multiple categories, (3) the linkage identified between the trait and FCB, and (4) any species or taxa mentioned in reference to the trait of interest. No new traits were extracted after screening around half of the studies, indicating saturation had been reached. However, repeated traits were recorded each time they appeared in the literature since additional publications provided further detail regarding the linkages between traits and FCB. See SI 1.1 for the systematic literature review protocol, SI 1.3 for inclusion/exclusion criteria, and SI 3 for additional details about strategies to collect and calculate trait data.

The relevant biological and ecological traits identified from the literature were grouped into different “themes” such as “Nutrition”, “Invasiveness”, “Climate change mitigation” and “Benefits to local biodiversity” (Fig. 3). These themes represent high-level topics under food security, climate change, and biodiversity (SI 1.4, Figure S1). Themes such as “Production efficiency” and “Environmental tolerance” overlapped across FCB categories, therefore linking traits with multiple FCBs. Trait association with food security focused mainly on the production potential and availability of seafood and how these themes are affected by issues of climate change and biodiversity. Many papers linked biological and ecological traits of aquaculture species with food production, which was then either explicitly or implicitly linked with food security.

### Developing the fuzzy expert system

We integrated aquaculture traits from the systematic literature review into a fuzzy expert system to quantify the potential of aquaculture species to contribute to food security, climate change and biodiversity (FCB) goals. The fuzzy system consists of (1) fuzzification, (2) fuzzy reasoning, and (3) defuzzification^36^, which are described below.

### Fuzzification

**Fig. 4:**
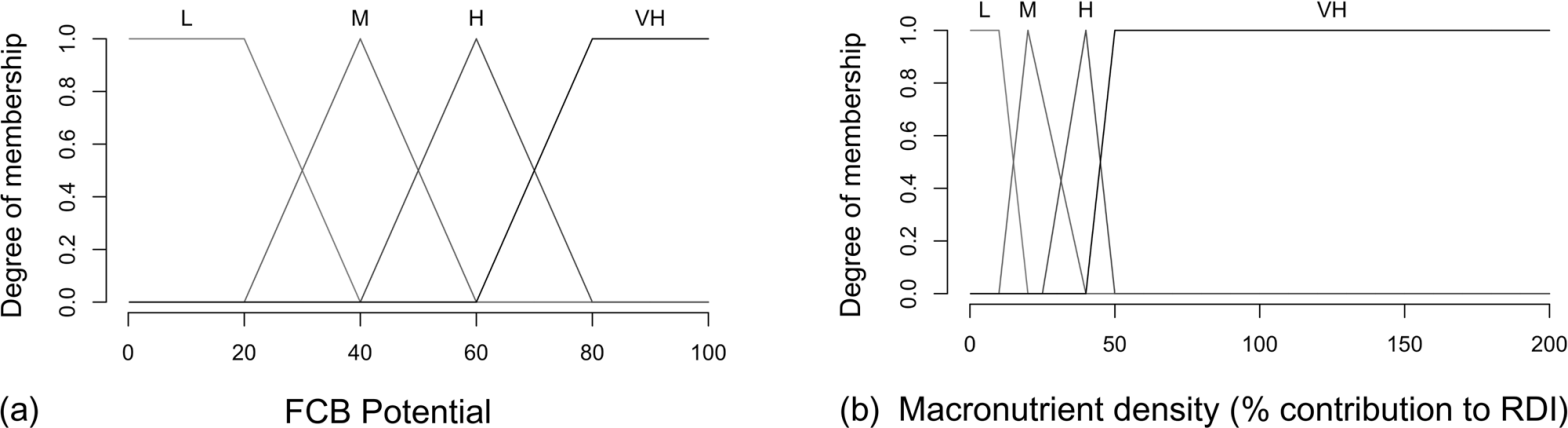
Fuzzy membership function examples. Figure 4 **a** shows output fuzzy sets for the FCB potential of aquaculture species, with potential scaled from 1 to 100. Some traits had opposite effects on FCB outcome (high aggression = low food security potential). Figure 4 **b** shows the fuzzy membership functions for one trait (macronutrient density) used in our expert system (L Low, M Medium, H High, VH Very high, RDI Recommended Daily Intake). See Figure S2 for membership functions for all traits. See Table S13 for corresponding FCB fuzzy sets for all traits.

Fuzzification is the process by which we used fuzzy membership functions to determine the degree of membership of trait values to each linguistic category. For every trait there are four linguistic categories (each is a “fuzzy set”), with corresponding membership functions (Fig. 4). Overlapping fuzzy sets allow species to be assigned to multiple sets simultaneously at membership levels defined by a fuzzy membership function.

We used trapezoid membership functions for the *low* and *very high* categories and triangular functions for *medium* and *high* categories^36^. The thresholds of the fuzzy sets were informed by existing knowledge of the relationship between traits and FCB. Lower and upper quartiles of traits (25th and 75th percentiles, respectively) were also used to calculate the fuzzy sets (low, medium, high, and very high categories). The amount of overlap, or “fuzziness”, between the categories indicates the uncertainty in assigning the level of potential. Trait values could belong to more than one fuzzy set with different levels of membership to each category defined by the membership functions in Fig. 4 and Figure S2, as long as the membership level exceeded the threshold value of 0.2 (ex: value for body size classified as *small*, *medium* and *large*). Missing trait data was penalized by assigning the value to the ‘low potential’ fuzzy set for the trait.

### Fuzzy reasoning (rule-firing)

The process of fuzzification transforms a set of input data into a range of conclusions using heuristic rules following the format:

If *A* then *B*, where *A* is the premise and *B* is the conclusion.

We used heuristic rules developed from relationships described in the literature that linked traits with FCB potential (Table S13). Rules were fired when the membership level exceeded a threshold value of 0.2. The degrees of memberships from each trait are traced to their corresponding contribution set. Species were classified into four linguistic categories of FCB potential (low, medium, high, very high) as a unique amalgamation of their attributes (Fig. 4a; Table S13 for corresponding FCB fuzzy sets for all traits). The expert system estimated the degree of membership to each linguistic category for each species.

We used an equal weighting of 0.5 for all rules, which corresponds to a 50% level of belief in the validity of the rule. Thus, the conclusion of any rule can have a maximum degree of membership of 0.5 to its fuzzy set. A minimum membership of 0.2 was set as the threshold value for rule-firing, based on the threshold used to omit conclusions with very low levels of membership in Cheung et al. ^36^.

Since the food, climate, and biodiversity potential of species were evaluated on the basis of multiple traits, we combined conclusions using an accumulation algorithm called MYCIN to obtain the final degree of membership to each of the four linguistic categories^101^. The following algorithm would calculate the final degree of membership associated with each level of conclusions (see Cheung et al. ^36^):

$${{\rm{AccMem}}}_{\left({\rm{i}}+1\right)}={{\rm{AccMem}}}_{\left({\rm{i}}\right)}+{{\rm{Membership}}}_{\left({\rm{i}}+1\right)}\left(1-{{\rm{AccMem}}}_{\left({\rm{i}}\right)}\right)$$

where AccMem is the degree of membership to a fuzzy set (e.g., to low food security potential) of conclusions from trait *i* or after combining conclusions from multiple traits.

*Membership*(i+1) denotes the membership of the trait being accumulated into the final degree of membership.

### Defuzzification

Defuzzification is the process of converting a fuzzy output (in this study, the four accumulated membership values to low, medium, high, and very high FCB potential) into a single ‘crisp’ value. We used defuzzification to calculate the final food security, climate change, or biodiversity contribution potential score for each species between 1 and 100. A score of 100 represents the highest potential to support F, C, or B. Each contribution potential category (x) corresponds to a defuzzification index value (*Ind*): Low = 1, Medium = 25, High = 75, and Very high = 100. These index values were weighted by their accumulated membership values (informed by the trait values) and then averaged to obtain the final food security, climate change, or biodiversity contribution potential score, *FinInd* (Cheung et al. ^36^):

$${FinInd}=\frac{\mathop{\sum }\limits_{x=1}^{n}{{AccMeM}}_{x}\times {{Ind}}_{x}}{\mathop{\sum }\limits_{x=1}^{n}{{AccMem}}_{x}}$$

See SI 4.2.1 for an example calculation of an FCB score using MYCIN and the above equation.

### Case studies

We applied the fuzzy logic framework to 54 major aquaculture species with trait data available on Fishbase or SealifeBase. These 54 species were identified from the Food and Agriculture Organization’s (FAO’s) 2022 State of World Fisheries and Aquaculture^2^, which lists the top-farmed inland and marine finfish, crustacean, mollusc, and algae species in 2020. This relatively small set of “staple” species comprises the vast majority of aquaculture production^2^. Proxies were used when species-level information was unavailable from FAO (Table S5; see SI 2 for more details about selecting species for analysis). Data for certain traits were missing on Fishbase and Sealifebase for some species, especially non-fish species. In these cases, trait data were collated from the literature.

To illustrate the application of the trait-based framework to describe the FCB status and priorities in more localized contexts, we compared the scores of three species with contrasting biological traits: Atlantic salmon (*Salmo salar*), Pacific oyster (*Magallana gigas*) and Sugar kelp (*Saccharina latissima*). Atlantic salmon and Pacific oyster are amongst the most economically important aquaculture species (marine and brackish water) by weight and value, including in British Columbia (B.C.), Canada^102–104^. Specifically, both species were introduced to B.C. for aquaculture, and negative impacts on biodiversity have been documented^46,47^. Seaweed aquaculture was suggested to hold large potential to meet FCB goals^105^. In North America, there has been recent interest and industry growth for sugar kelp (*Saccharina latissima*) aquaculture^106,107^, though its production still represents less than 0.001% of global algae aquaculture^2^. Sugar kelp is a close relative of Japanese kelp (*Saccharina japonica*), the highest-cultivated algae species globally^2^, which has demonstrated contributions to food security in Asia^108^ and the potential to sequester carbon^71,72^. Unlike Japanese kelp, however, sugar kelp is native to British Columbia waters^109^, making it an ideal species to consider for local aquaculture production. We use more readily available data for Japanese kelp to estimate the FCB potential of sugar kelp in this analysis. To see example calculations with explanations of the operation of the fuzzy logic framework, see SI 4.2.

### Sensitivity analysis

A jackknife analysis was used to evaluate the sensitivity of the system to our inputs and rules for determining FCB potential. We examined the effects of individual traits on the outputs of the fuzzy system through “jackknife resampling”, or repeating calculations through the sequential removal of one or more trait(s) at a time^110^ from a given set of attributes (F, C, or B). This analysis allowed us to assess the influence of each trait on the system output. Removal of traits that resulted in relatively large output deviations was considered to have a greater influence on the system. We then used the jackknife approach to calculate the FCB potential for the 54 major aquaculture species and compare the output of the system when an increasing number of traits were excluded. Specifically, we first used the entire set of input variables (n), then removed one to n-1 variables and their associated rules. We repeated these calculations 50 times to obtain a distribution of the deviations and evaluated the sensitivity of the index to varying levels of data availability by comparing the FCB potential after each trait removal to its baseline score (all traits included). See SI 5 for more details.

### Supplementary Information

Supplementary Information

## Data Availability

The code for this study is available at: https://github.com/aleahwong/aquaculture_FCB. A full list of studies included in the systematic review with extracted data is provided in the Supplementary Information (SI 1.5). Production data for major aquaculture species was obtained from the Food and Agriculture Organization’s 2022 State of World Fisheries and Aquaculture (10.4060/cc0461en). Species trait data are from FishBase (http://www.fishbase.org/), SealifeBase (http:// www.sealifebase.org), and the additional sources provided in SI 3.7. Invertebrate nutrition data were obtained from FAO/INFOODS Global Food Composition Database for Fish and Shellfish Version 1.0 (PDF; Excel: xlsx). Temperature, salinity, dissolved oxygen concentration, and nutrient concentration data used to calculate species thresholds are from the World Ocean Atlas 2018 (https://www.ncei.noaa.gov/access/world-ocean-atlas-2018 /). Species occurrence data are from Ocean Biogeographic Information System (OBIS, http://www.iobis.org/), Global Biodiversity Information Facility (GBIF, http://www.gbif.org/), FishBase (http://www.fishbase.org/) and SealifeBase (https://www.sealifebase.org/).
