# Supplementary material for: A traits-based approach to assess aquaculture’s contributions to food, climate change, and biodiversity goals
Source: NPJ Ocean Sustain. 2024 May 31;3(1):30. doi: 10.1038/s44183-024-00065-7 (PMC11142914; doi:10.1038/s44183-024-00065-7)
Supplement: Supplementary file 1 — Supplementary Information [file 44183_2024_65_MOESM1_ESM.pdf]

Supplementary Information

Table of Contents

1. **Systematic literature review**
    - **1.1 Systematic review protocol (link to file)**
    - **1.2 PRISMA Documentation**
        - Table S1. PRISMA checklist (link to file)
        - Table S2. PRISMA flowchart (link to file)
    - **1.3 Inclusion and exclusion criteria**
        - Table S3. Inclusion criteria for publications to be included in the SLR
        - Table S4. Traits from the literature review which were excluded from the fuzzy expert system
    - **1.4 Coding and synthesizing data for FCB Themes**
    - Figure S1. Themes synthesized during systematic review
    - **1.5 References and data for systematic review**
        - **1.5.1** Studies included in systematic review (link to file)
        - **1.5.2** Data extraction from systematic review (link to file)

2. **Selecting species for analysis**
    - Table S5. Species proxies

3. **Calculations of input trait data**

3.1 Calculation of ranges for temperature, nitrate, phosphate, salinity and pO2

3.2 Calculation of geographic range (water area) and latitudinal range

- Table S6. Rules linking FAO area and latitudinal range with new linguistic categories for geographic range
- Table S7. Corresponding water area values in km² for each linguistic category of geographic range

3.3 Assignment of strength of spatial behavior

- Table S8. Keywords that describe the spatial behavior of aquaculture species and their corresponding multiplication factors

3.4 Micronutrient and macronutrient density

- Table S9. Average RDA of 5 micronutrients and 2 macronutrients for children under 5
- Table S10. Linguistic categories for micronutrient (a) and macronutrient (b) density, with corresponding percent contributions of each nutrient in 100 grams wet weight to RDA.

3.5 Taxonomic group-based traits

- Table S11. Trophic levels assigned to species (based on taxonomic groups) when information was unavailable in FishBase and SealifeBase
- Table S12. Linguistic assignments for 5 traits based on taxonomic groupings (1-Fish, 2-Crustaceans, 3-Molluscs, 4-Algae)

3.6 Reproductive frequency

3.7 Data References (link to file)

4. **Fuzzy expert system**

4.1 Fuzzy membership
Table S13. Trait fuzzy sets and corresponding FCB contribution potential (link to file)
Figure S2. Fuzzy membership functions for traits used in our expert system
4.2 Example calculations
4.2.1 Example calculation of a species' final degree of membership
4.2.2 An illustrative example
4.3 Non-parametric species rankings
Table S14. Non-parametric species rankings (link to file)
4.4 Statistical tests on non-normal distributions of FCB scores
Table S15. Shapiro-Wilk normality test results
Table S16. Kruskal-Wallis test results
Table S17. Kruskal-Wallis multiple comparison using post-hoc Dunn's test

5. **Sensitivity analysis**
Figure S3. Deviations in index values from the baseline estimate (all traits included) of food security (a, b), climate change (c, d), and biodiversity (e, f) following removal of traits as inputs to the fuzzy system

---

1. Systematic literature review

We chose a qualitative review because our aim was to assess the *potential* of aquaculture species to contribute to FCB goals, and we felt this required an inclusive approach to deriving associations, one that would use existing knowledge to tell us about species' potential, without requiring absolute certainty about the strength of the relationship between traits and FCB outcomes. For our systematic review, we searched for qualitative descriptions of traits and their associations with FCB, and used words and text (anywhere from a sentence to a paragraph) to summarize and explain our findings. Text is often used as raw data for knowledge-building in qualitative systematic reviews[36]. Associations between traits and FCB did not always appear in the literature as primary findings, and did not depend on study design--components that are evaluated during a critical appraisal of *quantitative* studies[37]. Identifying trait-FCB associations involved making some inferences about the relationships of traits with FCB. For example, if one source mentioned that filter-feeding provides a "regulating service" by reducing eutrophication through uptake of excess nutrients, and another source mentioned filter-feeding along with the key word "biodiversity", then we were able to infer a linkage between the trait and biodiversity using multiple observations.

Inferences are common in both qualitative and quantitative research, and King et al. (1995) argue that "uncertain inferences are every bit as scientific as more certain ones so long as they are accompanied by honest statements of the degree of uncertainty accompanying each conclusion" [38]. We do not search for or state the strength of the relationship between traits and FCB using information collected in the review, as there can be inconsistency and uncertainty in trait values and FCB outcomes--making associations based on a broad range of sources with varying data availability tricky to derive. Rather, we use the fuzzy logic framework to explicitly include ranges in uncertainty, and we validate our results using appropriate techniques for qualitative reviews such as saturation and fit[39], which refers to the way findings are linked and interact to form a whole picture[40]. We kept track of new insights using a saturation curve, and achieved fit

by “filling in the blanks” as we generated understanding about how traits overlap across FCB categories and interact via cross-cutting FCB themes. That fit is a key indicator of validity in qualitative reviews also demonstrates the suitability of this approach to our research at the intersection of food, climate and biodiversity.

1.1 Systematic review protocol

SLR Protocol

1.2 PRISMA Documentation

Table S1. PRISMA checklist
PRISMA_2020_checklist.pdf
Table S2. PRISMA flowchart
PRISMA flowchart

1.3 Inclusion and exclusion criteria

Table S3. Inclusion criteria for publications to be included in the SLR

**Criteria**	**Decision**
When the predefined keywords exist in the specified section (title, abstract, text)	Inclusion
The paper is available in English	Inclusion
The paper is published in a scientific peer-reviewed journal or is a form of gray literature such as a government report, dissertation or conference paper	Inclusion
The paper is a study focused on species which are used in aquaculture	Inclusion
The paper describes functional aquaculture traits	Inclusion
The paper address at least one of FCB as a challenge, goal, opportunity, etc.	Inclusion
Papers published between 2000 and 2023	Inclusion

Excluded traits

We refined the list of traits by selecting explicit, functional traits for which data could be found. Since differing production and environmental contexts including genetic modification, pollution, recirculation tanks, and novel feeding strategies can alter the FCB potential of species[12, 34, 35] and are hard to control for when analyzing a broad range of species, we chose attributes that are

inherent to a species. Our assumption during analysis was that all species were subject to the same conditions. Thus, the inherent traits revealed more about the actual species' FCB potential and less so about aquaculture technology. Very broad characteristics such as "complexity of life cycle" were broken down into more specific attributes, while very specific traits were grouped into attributes of a more descriptive and appropriate scale. The final list of traits (Figure 1) was used to create the fuzzy expert system.

The following traits which appeared in the literature search were excluded from the fuzzy expert system because they were not explicit functional traits:

Table S4. Traits from the literature review which were excluded from the fuzzy expert system

FCB Category	Omitted trait	Reason for exclusion
Food security	Mode of fertilization (internal vs. external)	Nested under fecundity
Food security	Spawning strategy (broadcast; passive)	Nested under fecundity
Food security	Toxicity	Context-dependent
Food security	Nutrient bioavailability	Context-dependent; data-poor
Food security	Ability to synthesize bioactive compounds	Link to food security unclear; too specific
Food security	Juvenile and adult survival	Context-dependent
Food security	Age at maturity	Nested under growth; beneficial for some species, detrimental for others
Climate change	Complexity of reproductive cycle (larval requirements)	Too broad; many definitions for complexity; larval stage not relevant for every taxa
Climate change	Oxygen and water requirements	Oxygen requirement included through inclusion of pO2 range; water requirement is context-dependent
Climate change	Habitat and prey specificity	Nested under geographic range; prey specificity related to feed efficiency/trophic level
Biodiversity	Native vs. non-native	Location-dependent

Biodiversity	Symbioses with micro-organisms	Context-dependent, data poor
FCB	Disease resistance	Often genetically modified; context-dependent

1.4 Coding and synthesizing data for FCB Themes

Understanding the relationship between species traits and ecosystem functioning is crucial before using traits as indicators[1]. Synthesis allowed us to refine our definitions of food security, climate change and biodiversity goals and improve our understanding on how traits were related to these goals. We coded the data extracted from each of the 152 publications into four categories: food security, climate change, biodiversity, and "interconnected", for traits that fit multiple categories. Traits in the latter category also helped explain the relationship between F, C and B categories.

Based on the traits that were grouped together, we were able to synthesize themes or "desirable outcomes" for each FCB goal. These "desirable outcomes" bolster the FAO definitions of food security, climate change and biodiversity and further contextualize the importance of species' biological and ecological characteristics. Desired outcomes for food security include consistent, accessible, and efficiently produced aquaculture to support the long-term needs of communities[2, 3, 4, 5, 6]. Aquaculture can contribute to both direct and indirect forms of food security by providing nutrition, as well as income[7]. According to the literature, desired outcomes for aquaculture related to climate change are adaptation to global change and reduction of aquaculture's environmental footprint for resilient production[8, 9, 10, 11]. The desired biodiversity outcome of aquaculture was the protection of wild, especially native species and ecosystems through resource use efficiency and reducing dependency on wild stocks for feed[12, 13, 14, 15].

By organizing traits under these themes, the linkages between traits and desired FCB outcomes became clear. For example, traits associated with food security were generally related to a species' suitability for mass culture under variable environmental conditions, nutritional density and resource use efficiency. Traits associated with climate change had to do with a species' environmental robustness, efficiency in producing nutrition, and its climate change mitigation potential. For biodiversity, traits usually indicated the risk of species becoming invasive as well as the biodiversity footprint of its resource use and potential to improve environmental quality.

For traits that were coded as "interconnected", synthesis yielded three major themes about how issues of food security, climate change and biodiversity are related. These themes helped to contextualize traits that appeared in multiple FCB categories. Traits associated with both food security and climate change revealed the goal of "producing accessible, efficient nutrition in a changing world", while traits linked to both food security and biodiversity revealed the importance of "producing nutrition with minimal environmental impacts". A third theme, "synergies and trade-offs between climate change and biodiversity" emerged from traits in the respective categories (Figure S1). Synthesized themes and definitions acknowledge the interconnectedness of food security, climate change and biodiversity.

We refined the list of traits by selecting explicit, functional traits for which data could be found. Since differing production and environmental contexts including genetic modification, pollution, recirculation tanks, and novel feeding strategies can alter the FCB potential of species [12, 34, 35] and are hard to control for when analyzing a broad range of species, we chose attributes that are

inherent to a species (See Table S4 for list of omitted traits). Our assumption during analysis was that all species were subject to the same conditions. Thus, the inherent traits revealed more about the actual species' FCB potential and less so about aquaculture technology. Very broad characteristics such as "complexity of life cycle" were broken down into more specific attributes, while very specific traits were grouped into attributes of a more descriptive and appropriate scale. The final list of traits (Figure 1) was used to create the fuzzy expert system. See Supplementary Information 3 for additional details about strategies to collect and calculate trait data.

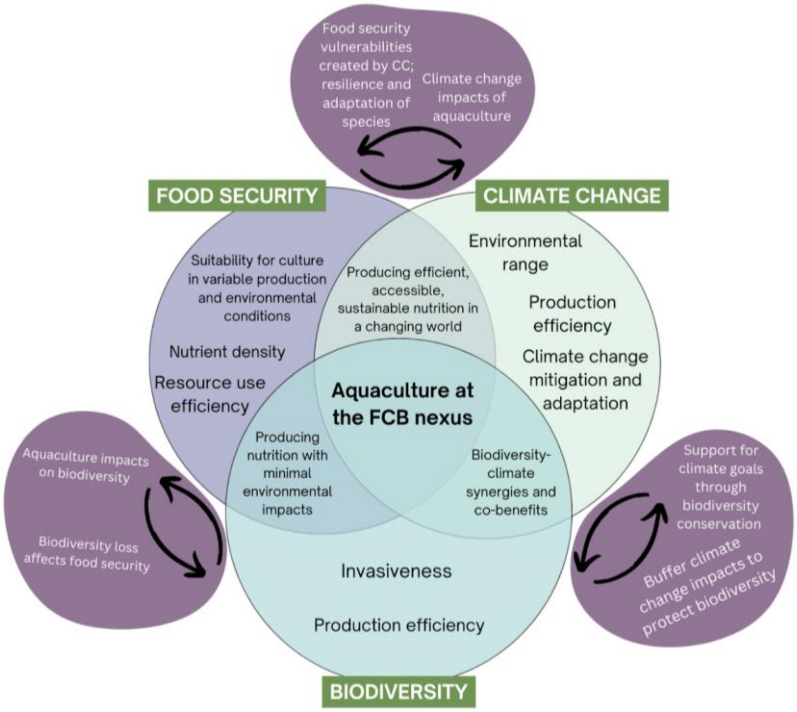

Figure S1. Cross-cutting FCB themes synthesized from traits collected during the systematic literature.

1.5 References and data for systematic review

1.5.1 References for studies included in systematic review: SLR references

1.5.2 Data extraction from systematic review: SLR_data.xlsx

2. Selecting species for analysis

We analyzed 54 major aquaculture species (by production) listed by the Food and Agriculture Organization (FAO) in the 2022 State of World Fisheries and Aquaculture[14]. The FAO lists the 15 most-farmed inland and coastal/marine finfish species, and the top eight farmed crustacean, mollusc and algae species. This relatively small set of "staple" species comprise the vast majority of aquaculture production[14]. For example, the top 15 farmed inland finfish species represent 79.3 percent of the global inland aquaculture production in 2020 and grass carp by itself accounts for 11.8 percent percent of production in the same category. In marine and coastal aquaculture (2020), Atlantic salmon makes up 32.6 percent of global production. This percentage rises to 77 percent when 14 other major marine and coastal finfish species are considered. Eight

major species are responsible for a high 95.3 percent of crustacean production, with whiteleg shrimp representing the largest proportion of 51.7 percent. In mollusc aquaculture, 84 percent of production is driven by eight species including cupped oysters (30.7 percent) while algae production (93.7 percent) is dominated by just eight species, led by Japanese kelp (35.5 percent). Since Nile tilapia and rainbow trout were included as major species in both inland and marine/coastal production, we used distinct annual production values to weight their FCB potential scores.

FAO reports data for "species items" including individual species, finfish hybrids, and groups of species identified at the genus, family or higher levels[14]. Rather than omitting highly-produced species from analysis, we opted to use species proxies when data were only available at the genus level or higher (See Table S1). To find reasonable proxies, we chose commonly farmed species that appeared on the FAO Aquatic Species Fact Sheets after searching for the genus or family listed on FAO aquaculture species database (https://www.fao.org/fishery/en/culturedspecies/search). This resulted in 54 unique species across four broad taxonomic groups (finfish, crustaceans, molluscs, algae). We used the species-level proxies to search for trait data on Fishbase and SealifeBase.

Table S5. Species proxies used for analysis when data were only available at the genus or higher taxonomic classification.

**Category**	**SOFIA entry**	**Proxy**
Inland finfish	*Carassius* spp.	*Carassius carassius*
Inland finfish	*Clarias* spp.	*Clarias gariepinus*
Marine finfish	*Epinephelus* spp.	*Epinephelus coioides*
Marine finfish	*Mugilidae*	*Mugil cephalus*
Molluscs	*Crassostrea* spp.	*Crassostrea gigas*
Molluscs	*Crassostrea* spp.	*Crassostrea virginica*
Molluscs	*Pectinidae*	*Mizuhopecten yessoensis*
Molluscs	*Mytilidae*	*Mytilus edulis*
Molluscs	*Mytilidae*	*Mytilus galloprovincialis*
Molluscs	*Mytilidae*	*Perna canaliculus* (*Mytlius canaliculus*)
Algae	*Eucheuma* spp.	*Eucheuma denticulatum*

Category	SOFIA entry	Proxy
Inland finfish	*Carassius* spp.	*Carassius carassius*
Algae	*Gracilaria* spp.	*Gracilaria gracilis*
Algae	*Porphyra* spp.	*Pyropia tenera* (formerly *Porphrya*)
Algae	*Porphyra* spp.	*Porphyra haitanensis*
Algae	*Porphyra* spp.	*Pyropia yezoensis* (formerly *Porphyra*)

3. Calculations of input trait data

Most biological and ecological trait data were available on Fishbase and SealifeBase for the 54 analyzed species. Information for some traits were not included in either database, and non-fish species in particular lacked data on traits such as growth rate and fecundity. When key traits associated with FCB were not available in Fishbase or SealifeBase we calculated values for each species based on information available in additional databases such as the Ocean Biodiversity Information System (OBIS), Global Biodiversity Information Facility (GBIF), World Ocean Atlas (WOA18) and datasets from the Nereus Program.

To find environmental range data we used published methods and species occurrence data to calculate plausible species tolerances to pO2, temperature, nitrate, phosphate, salinity as well as their latitudinal range and water area occupied. Lower and upper quartiles of each trait (25th and 75th percentiles, respectively) were used to calculate the fuzzy ranges (low, medium, high and very high overlapping categories). Lack of data was penalized. When no trait data were published for a species and calculations could not be performed to elucidate trait information, the species was assigned to the "low potential" linguistic category for the attribute. Unless otherwise specified, we used quartiles of each trait's data distribution (25th, 50th, 75th percentiles) to inform the fuzzy set thresholds.

3.1 Calculation of ranges for temperature, nitrate, phosphate, salinity and pO2

To calculate species ranges for temperature, nitrate, phosphate and salinity, we compared global species occurrence data from GBIF and OBIS with WOA18 data for monthly averages of the four variables. Ranges were defined as the minimum (first percentile) and maximum (99th percentile) values for each ocean variable where the species can be found.

While dissolved oxygen in seawater has often been used to represent a species' oxygen range, the partial pressure of O2 (pO2) in seawater provides a more nuanced indicator of where a species can live. This is because oxygen content in water is highly influenced by temperature, and pO2 can reveal temperature-dependent O2 thresholds[16, 17]. Species-specific ratios between pO2 supply and demand, also known as the Aerobic Growth Index (AGI)[16], can also be used as an index of habitat viability[17]. With the formula outlined in Morée et al. (2022), we used salinity, temperature (mean of sea surface and 200 meters) and dissolved oxygen data (WOA18)

to calculate the partial pressure of oxygen in seawater. We then used species occurrence data from GBIF and OBIS to find global species pO2 thresholds, defined as the pressure (in mbar) at which 10 percent of the species data occur (10th percentile).

3.2 Calculation of geographic range (water area) and latitudinal range
Geographic range in terms of the area of the ocean surface (km²) corresponding with where a species lives, as well as latitudinal range, reveal how broadly a species can live. To calculate spatial ranges, we opted to use Fishbase species distribution data which are inputs to the dynamic bioclimate envelope model (DBEM) developed by Cheung et al. (2008)[18]. Using ocean surface area data on a 0.5° × 0.5° resolution grid, we summed the area in each cell where a given species had distribution or occurrence data to find the amount of viable ocean area in square kilometers for each species. Minimum and maximum latitudes of each species' distribution were used to find the latitudinal range.

Species distributions are much larger than represented by occurrence data from GBIF and OBIS, but not all of our species are included in the distribution data. For those species lacking distribution information, we substituted occurrence data from OBIS to find latitudinal range and developed a scheme to calculate geographic range based on latitude information and FAO area data. Using Fishbase and SealifeBase, we found the total shelf area of the FAO areas each species is native to. We then calculated the percentage of inland waters and/or ocean areas in the species' range based on the FAO shelf area. Using rules that take into account both latitudinal range (from OBIS) and ocean/inland percentage (Table S2), we classified species into a preliminary fuzzy set (linguistic category from low to very high geographic range) which was then translated into a new value (in km²) that placed the species at the appropriate level (low to very high) amongst the other species which had distribution data (Table S3).

We used the following rules to transform latitudinal range and FAO area into geographic range (water area):

Table S6. Rules linking FAO area and latitudinal range with new linguistic categories for geographic range. 60° was chosen as the latitudinal range threshold between linguistic categories since it is the mean latitudinal range.

IF total FAO area % is:	AND latitudinal range is:	Geographic range is:
0-30%	< 100°	LOW
0-30%	≥ 100°	MEDIUM
30-50%	< 60°	LOW
30-50%	≥ 60°	MEDIUM
50-70%	< 60°	MEDIUM
50-70%	≥ 60°	HIGH

70-100%	< 60°	HIGH
70-100%	≥ 60°	VERY HIGH

Table S7. Corresponding water area values in km^2 for each linguistic category of geographic range.

**Linguistic category (Geographic range)**	**Assigned water area value (km^2)**
Low	100,000
Medium	2,000,000
High	7,000,000
Very High	15,000,000

3.3 Assignment of strength of spatial behavior

To assign aquaculture species a score for spatial behavior, we looked for keywords in Fishbase and SealifeBase that describe the spatial behavior of species (Table S4) and followed the method in Cheung et al. (2005) which assumes a baseline spatial behavior strength of 1 for species forming groups, 40 for species forming aggregations or shoals, and 80 for schools[19]. We then multiplied the baseline spatial behavior strength (*B*) by adjustment factors (*A*) determined by the linguistic descriptions available for each species:

$$S = B \times (1 + A_1 + A_2 + \ldots A_n)$$

S is the total spatial behavior strength between 1 and 100 and *n* is the number of keywords included.

Due to their high population densities, sessile species such as those belonging to mollusc and algae taxonomic groups were assigned high spatial behavior scores (> 60) that placed them in the high and very high categories.

Table S8. Keywords that describe the spatial behavior of aquaculture species and their corresponding multiplication factors

**Keywords**	**Multiplication (*A*)**
Usually solitary/pair	-40%
Occasionally/sometimes/alternately/may/probably/loose/small	-40%
Sometimes solitary/pair	-20%

Presumably/apparently	-20%
Frequently/often	20%
Commonly/usually/large/dense	40%

3.4 Micronutrient and macronutrient density

We extracted species data for protein and omega-3 polyunsaturated fatty acids (grams per 100 grams) from Fishbase and Sealifebase. Protein and fats are oftentimes the only nutrients included in food security indices, despite the importance of micronutrient intake for adequate nutrition[20]. We used data from Fishbase for calcium, iron, selenium, vitamin A and zinc densities, which are crucial for human health yet have low levels of adequate intake globally[21, 22]. Following Maire et al. (2021), we defined micronutrient density as the percent contribution of 100 grams of wet weight to the recommended dietary allowance (RDA), the daily intake level that meets the dietary needs of 97-98% of the population[23, 41]. While we used the average RDA for children under 5 years old (Table S5), our results are likely to apply across age groups because recommended dietary allowance between the population group we used and the rest of the population are strongly correlated[24]. The percent RDA contribution of each of the 5 micronutrients in 100g of wet weight were summed together with a maximum of 100% per nutrient, for a maximum of 500% RDA total. Capped percentage contributions to RDA prevent extreme values from skewing the variation in micronutrient density scores.

We consulted the Federal Drug Administration’s Percent Daily Value index to help inform the fuzzy sets (low, medium, high, very high) we used to classify species based on nutrient density (Table S6). Percent Daily Value indicates whether a serving of food is high (20 percent or more) or low (5 percent or less) in a particular nutrient[25].

Table S9. Average RDA of 5 micronutrients and 2 macronutrients for children under 5 (6 months to 5 years, since < 6 months assumed to be breastfed). We used a conservative bioavailability of 5% for iron to reflect a broad population which may include those with limited consumption of vitamins that increase the bioavailability of iron, those consuming plant-based sources of iron with lower bioavailability rates, and those with low iron stores. Pre-existing iron status has been shown to greatly influence further absorption of iron[25].

Vitamin A	280 μg
Calcium	560 mg
Iron	14.3 mg at 5% bioavailability
Selenium	16.33 μg
Zinc	4.33 mg
Omega-3 fatty acids	0.7 g

Protein	14.3 g

Table S10. Linguistic categories for micronutrient (a) and macronutrient (b) density, with corresponding percent contributions of each nutrient in 100 grams wet weight to RDA.
(a)

**Linguistic category (*micro*nutrient density)**	**Percent contribution of 100g wet weight to RDA**
LOW	50% ≥ ; each micronutrient ~ < 10% RDA
MEDIUM	25% < MD < 100%; Each micronutrient ~ 5-20% RDA
HIGH	75% < MD < 120%; Each micronutrient ~ 15-25% RDA
VERY HIGH	100% ⩽ MD; each micronutrient ≥ 20%

(b)

**Linguistic category (*macro*nutrient density)**	**Percent contribution of 100g wet weight to RDA**
LOW	20% ≥ ; each macronutrient ~ < 10% RDA
MEDIUM	10% < MD < 40%; Each macronutrient ~ 5-20% RDA
HIGH	25% < MD < 50%; Each macronutrient ~ 12-25% RDA
VERY HIGH	40% ⩽ MD; each macronutrient ≥ 20%

Nutrient data were more difficult to find for non-fish species. We used unpublished micronutrient data for invertebrate species calculated by co-author Christina Hicks. For algae species we found macro- and micronutrient data from the literature and converted dry weight nutrient content into wet-weight values[27, 28, 29].

3.5 Taxonomic group-based traits

Some traits like trophic level, pH sensitivity, and carbon sequestration, ocean acidification buffering, bioremediation and structural provisioning potential were assigned based on each species' taxonomic group. When trophic level was not available in FishBase or SealifeBase, the following assignments were made:

Table S11. Trophic levels assigned to species (based on taxonomic groups) when information was unavailable in FishBase and SealifeBase

1	Algae
1.5	Filter-feeders (ex: bivalves)
2	Herbivores
2.5	Omnivorous
3	Carnivorous
4+	Apex predators

We grouped species into one of four broad taxonomic categories (1-Fish, 2-Crustaceans, 3-Molluscs, 4-Algae) to categorize their pH sensitivity and potential for carbon sequestration, ocean acidification buffering, bioremediation and structural provisioning. The rules in Table S8 are based on the literature[30, 31] and were used to assign species linguistic values for each of the 5 traits based on their taxonomic grouping. For example, we assumed high (membership = 0.25) to very high (membership = 0.75) sensitivity to PH for molluscs (row 1 of Table S8).
For finfish and algae, we assumed low (membership = 0.75) to medium sensitivity (membership = 0.25, and for crustaceans, we assumed medium (membership = 0.25), high (membership = 0.5) or very high sensitivity (membership = 0.25).

Table S12. Linguistic assignments for 5 traits based on taxonomic groupings (1-Fish, 2-Crustaceans, 3-Molluscs, 4-Algae).

**Trait**	**Low**	**Medium**	**High**	**Very High**
pH Sensitivity	(0.75, 0, 0, 0.75)	(0.25,0.25,0,0.**25**)	(0,0.5,0.25,0)	(0,0.25,0.75,0)
Carbon sequestration potential	(1, 1, 0.25, 0)	(0, 0, 0.25, 0.25)	(0, 0, 0.25, 0.5)	(0, 0, 0.25, 0.25)
Ocean acidification buffering potential	(1, 1, 1, 0)	(0, 0, 0, 0.5)	(0, 0, 0, 0.25)	(0, 0, 0, 0.25)
Bioremediation potential	(1, 1, 0.25, 0.25)	(0, 0, 0.5, 0.5)	(0, 0, 0.25, 0.25)	(0, 0, 0, 0)
Structural provisioning potential	(1, 1, 0, 0)	(0, 0, 0.50, 0.25)	(0, 0, 0.25, 0.25)	(0, 0, 0.25, 0.5)

3.6 Reproductive frequency

Arbitrary categories were created for reproductive frequency from “once/lifetime” to “several times/year” in order to accommodate species in every taxonomic category. “Perennial” algae

species were assigned to “year-round” while “annual” species were categorized as “once/year” under the new arbitrary levels.

3.7 Data References

[Data References](#)

4. Fuzzy expert system

4.1 Fuzzy membership

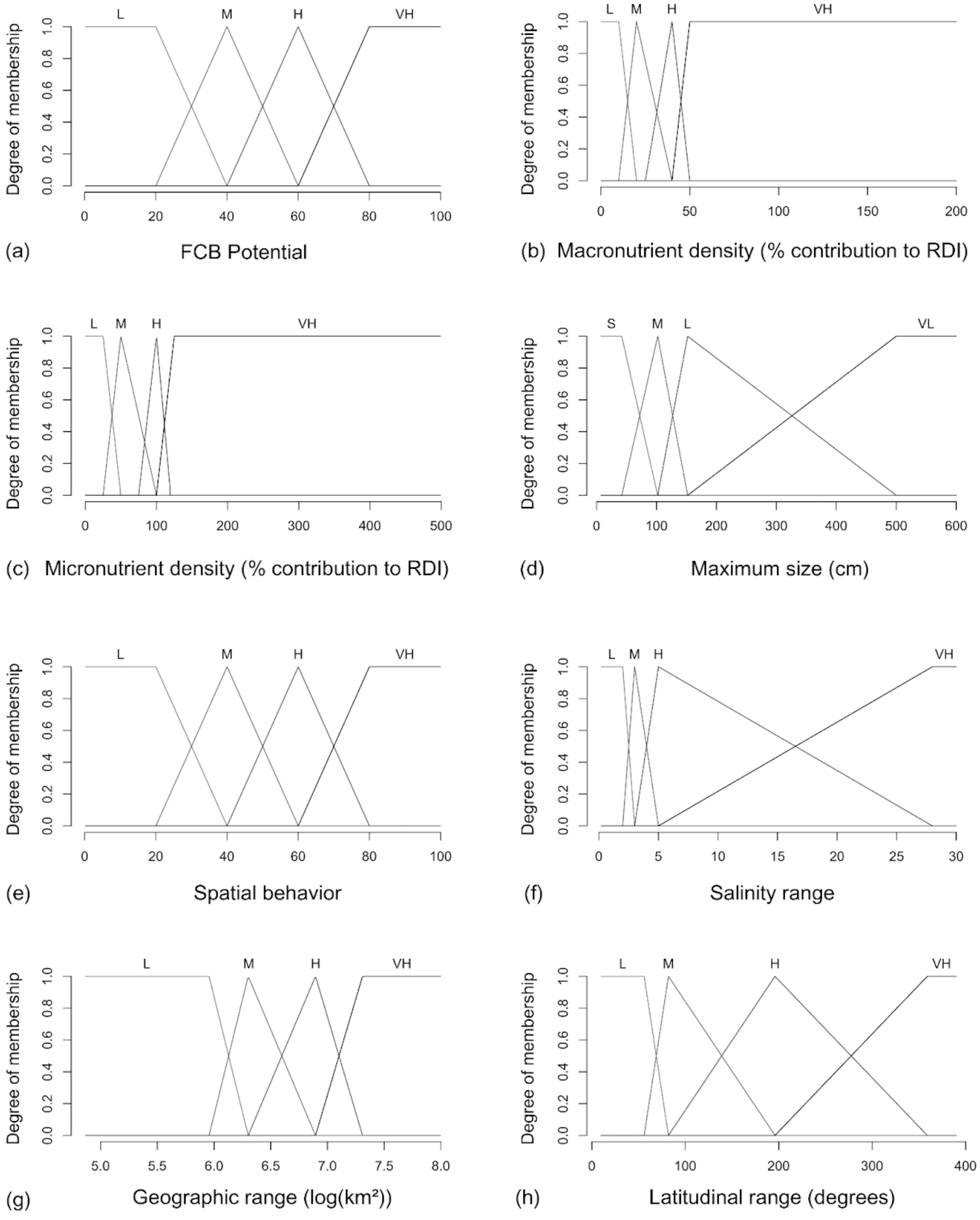

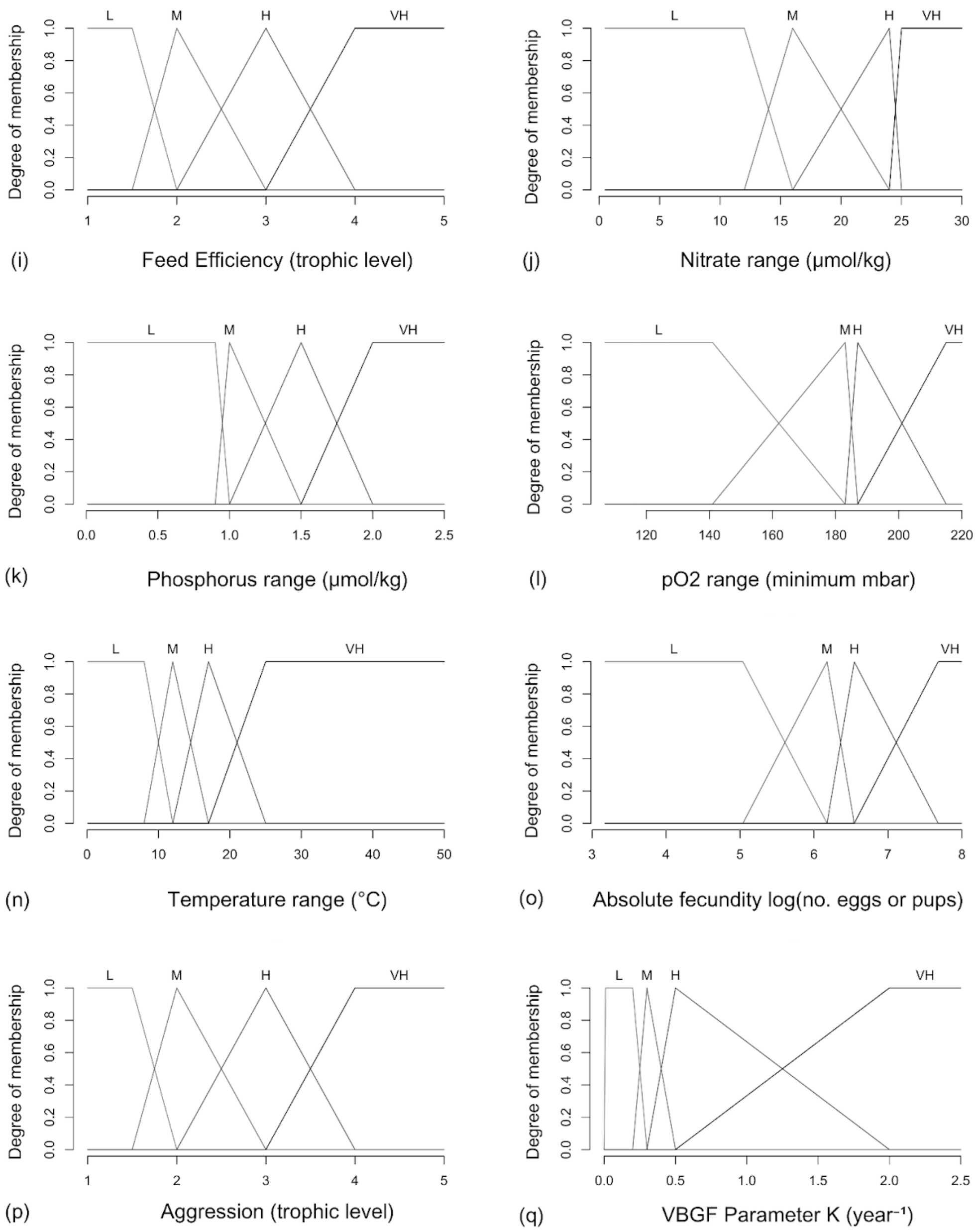

Figure S2. Fuzzy membership functions for traits used in our expert system (L = Low, M = Medium, H = High, VH = Very high). For maximum size (d), S = Small, M = Medium, L = Large, VL = Very large. **Graph a** shows output fuzzy sets for the FCB potential of aquaculture species, with potential scaled from 1 to 100. Some traits had opposite effects on FCB outcome (high aggression = low food security potential). Salinity range (f) is unitless. VGBF (q) stands for Von Bertalanffy Growth Function. The coefficient K describes how quickly a species can reach maximum length. We log-transformed the scale for absolute fecundity (o) and geographic range (g). Trophic level serves as a proxy for feed efficiency (i) and aggression (p). See Table S13 for corresponding FCB fuzzy sets for all traits.

Table S13. Trait fuzzy sets and corresponding FCB contribution potential
Corresponding fuzzy sets

4.2 Example calculations

4.2.1 Example calculation of a species' final degree of membership

If the fuzzy system assigned a species to the "low" food security fuzzy set for the von Bertalanffy growth factor K (VBGF K) and absolute fecundity with membership values of 0.26 and 0.58, respectively, and to the "medium" set for VBGF K (0.42) and macronutrient density (0.19), the MYCIN algorithm would calculate the final degree of membership associated with each level of conclusions using the MYCIN algorithm below[31] (see Cheung et al. 2005):

$$AccMem_{(i+1)} = AccMem_{(i)} + Membership_{(i+1)} \, (1 - AccMem_{(i)})$$

where AccMem is the degree of membership to a fuzzy set (e.g., to low food security potential) of conclusions from trait *i* or after combining conclusions from multiple traits. $Membership_{(i+1)}$ denotes the membership of the trait being accumulated into the final degree of membership.

To accumulate the "low" conclusions from the illustrative example above:

AccMem (VBGF K and absolute fecundity) = 0.26 + (0.58 x (1- 0.26)) = 0.69

To accumulate the "medium" conclusions from the illustrative example above:

AccMem (VBGF K and macronutrient density) = 0.42 + (0 x (1- 0.42)) = 0.42

Since the membership of the macronutrient density value to the medium contribution set (0.19) is smaller than the threshold of 0.2, the membership value is transformed to zero (Cheung et al. 2005).

The calculation to defuzzify the membership values from "low" and "medium" food security contribution potential categories would be:

FinInd= [(0.69x 1) + (0.53 x 25)] / (0.69 + 0.53) = 11.43 (this example conclusion is quite low because only the low and medium contribution levels were defuzzified).

4.2.2 An illustrative example

The fuzzy system calculated scores along three indices (food security, climate change and biodiversity potential) for 54 major aquaculture species. The example below shows the calculation of food security scores for Atlantic salmon, an economically important aquaculture species in British Columbia:

**Trait**	**Value**	**Fuzzy Set(s)**	**Food Security**

Aggression (trophic)	4.5	Low (0.5)	Low (0.96), Medium (0.76), High (0.74), Very high (0.81)
Spatial Behavior (unitless)	40	Medium (0.5)	**Final Food Security Index: 47.61**
Temperature Range (C)	13.19	Medium (0.38), High (0.11)	
pO2 Range (units)	107.29	Low (0.5)	
Nitrate Range (units)	12.88	Low (0.39), Medium (0.10)	
Phosphate Range (units)	1.98	High (0.02), Very high (0.48)	
VBGF (K)	0.44	Medium (0.15), High (0.35)	
Maximum Size (cm)	150	Medium (0.02), High (0.48)	
Feed Efficiency (trophic)	4.5	Low (0.5)	
Absolute Fecundity (# eggs)	26000	Low (0.5)	
Micronutrients (% RDA)	114.78	High (0.13), Very High (0.30)	
Macronutrients (% RDA)	200	Very high (0.5)	

Degree of membership is included in parentheses. Membership values < 0.2 were excluded from calculations (since a weighting factor of 0.5 was used, a membership value of 0.1 here represents a raw membership score of 0.2, the threshold level). The accumulated degrees of membership to each fuzzy set were Low (0.96), Medium (0.76), High (0.74), Very High (0.81) and the final food security index is 47.61.

Atlantic salmon has low potential to contribute to food security in terms of its trophic level (aggression, feed efficiency), pO2 range, nitrate range and absolute fecundity. These traits could be targeted to improve the food security potential of Atlantic salmon. By contrast, the VBGF (K), maximum size, nutrient density and phosphate ranges of Atlantic salmon indicate high food security potential. All traits considered, Atlantic salmon has a final food security index of 47.61, on a scale of 1 to 100.

4.3 Non-parametric species rankings

Table S14. Non-parametric rankings

4.4 Statistical tests on non-normal distributions of FCB scores

Table S15. Shapiro-Wilk normality test results. A p-value < 0.05 indicates the observations vary significantly from a normal distribution.

Index	p-value
Food security	p = 0.00916
Climate change	p = 0.4458
Biodiversity	p = 0.006199

Table S16. Kruskal-Wallis test results on the FCB scores. A p-value < 0.05 indicates that at least one taxonomic group has a significantly different median index from other groups.

Index	p-value
Food security	p = 8.215e-05
Climate change	p = 1.412e-06
Biodiversity	p = 7.019e-08

Table S17. Kruskal-Wallis multiple comparison using post-hoc Dunn's test. P-values were adjusted using the Holm method. A = Algae; C = Crustacean; F = Finfish; M = Mollusc

	A-C	A-F	C-F	A-M	C-M	F-M
F	0.00490	0.000665	0.714	0.807	0.0363	0.0177
C	7.078 e-07	1.514 e-04	4.160 e-02	1.019 e-02	2.846 e-02	6.233 e-01
B	2.895 e-02	2.874 e-05	5.509 e-01	7.566 e-0	1.589 e-02	5.372 e-06

5. Sensitivity analysis

Jackknife analysis showed that median deviations in the estimated indices for food security (Figure S3a), climate change (Figure S3c) and biodiversity (Figure S3e) were very small when individual traits were removed from the fuzzy system. Upper and lower quartiles (25% and 75%) of the deviations in indices were usually within 2 points (maximum of 100) relative to the baseline estimates when all traits were included for food security, and within 5 points for climate change and biodiversity.

The food security index was most sensitive to absolute fecundity, macronutrients, maximum size, phosphate range (Figure S3a). Phosphorus range was included as a trait in the food security fuzzy system because the presence of nutrients such as phosphorus and nitrates is common in high-density rearing environments and tolerance to high nutrient levels can be beneficial for production[33]. The deviations in the food security index following the removal of these traits are greater than 10 index points in one or both directions. Removal of absolute fecundity and maximum size tended to result in unsymmetrical negative bias on the predicted food security index. In contrast, excluding macronutrients or phosphate range tended to result in positive bias. The food security index was moderately sensitive to VBGF (K) and nitrate range. Removal of these traits resulted in deviations greater than 5, but less than 10 index points.

The climate change index was most sensitive to feed conversion efficiency (using trophic level as a proxy), nitrate range and sensitivity to pH, with deviations greater than 10 index points in either direction (Figure S3c). The exclusion of each of these three traits from the system resulted in a negative bias on the climate change index. Removing pH sensitivity resulted in deviations up to -20 compared to the baseline. The climate change index was moderately sensitive to the removal of phosphate range, pO_2 tolerance and salinity range.

The biodiversity index was most sensitive to absolute fecundity, latitudinal range, geographic range, and VBGF (K) (Figure S3e). Removal of each of these traits resulted in unsymmetrical negative bias on the biodiversity index, with deviations greater than 10. Deviations neared -20 when fecundity and VBGF (K) were removed from the system. In contrast, the removal of taxonomic-based traits such as bioremediation potential and structural provisioning potential resulted in a positive bias on the biodiversity index.

When we randomly removed an increasing number of attributes from the system the index value deviations of food security (Figure S3b), climate change (Figure S3d) and biodiversity (Figure S3f) increased compared with the baseline estimates. The medians of deviated index values for all three indices stayed around zero due to symmetrical deviations (positive and negative biases) following the removal of traits. However, maximum deviations increased to greater than -40 index points when four or more traits were excluded from index calculations of climate change and biodiversity (Figure S3d and S3f). Maximum deviations increased to greater than 20 points in either direction when three or more traits were excluded from index calculations of food security (Figure S3b).

When we randomly removed an increasing number of attributes from the system the index value deviations of food security, climate change and biodiversity increased compared with the baseline estimates (Figure S3). The medians of deviated index values for all three indices stayed around zero due to symmetrical deviations (positive and negative biases) following the removal of traits. However, maximum deviations increased to greater than -40 index points when four or more traits were excluded from index calculations of climate change and biodiversity (Figure S3d and S3f). Maximum deviations increased to greater than 20 points in either direction when three or more traits were excluded from index calculations of food security (Figure S3b).

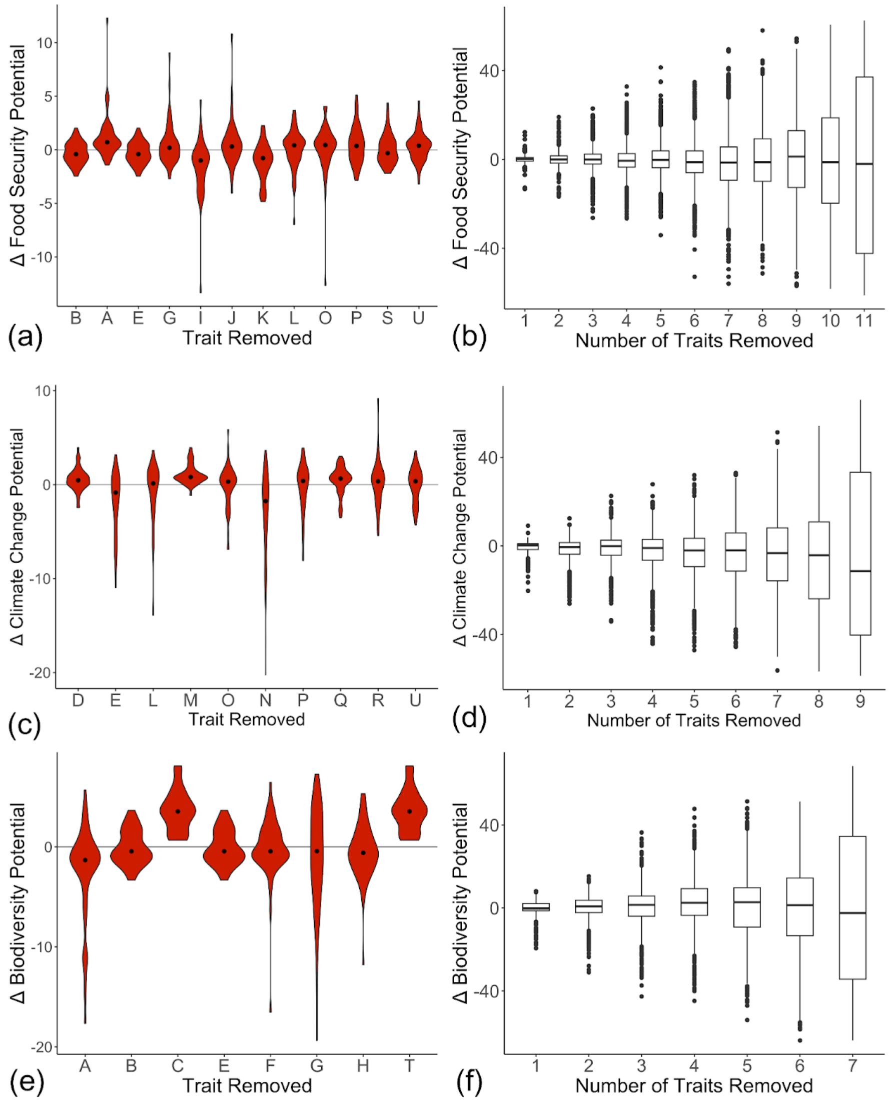

Figure S3. Deviations in index values from the baseline estimate (all traits included) of food security (a, b), climate change (c, d), and biodiversity (e, f) following removal of traits as inputs to the fuzzy system. The black dots (a, c, e) and band in the middle of the box (b, d, f) are the median of the deviations of the 54 aquaculture species when attributes were removed (a, c, e) and an increasing number of attributes were randomly excluded (b, d, f). Violin plots in (a, c, e) show the minimum, maximum and distribution of deviations. The bottom and top of the boxplots in (b, d, f) show the 25th and 75th quartiles of the deviations, respectively. The lower and upper end of each vertical line represent the minimum and maximum values, while the black dots are outliers. Traits removed in (a), (c) and (e) are: **A** (Absolute fecundity); **B** (Aggression--trophic level); **C** (Bioremediation potential); **D** (Carbon sequestration potential); **E** (Feed conversion efficiency--trophic level); **F** (Geographic range); **G** (VBGF K); **H** (Latitudinal range); **I** (Macronutrient density); **J** (Maximum size); **K** (Micronutrient density); **L** (Nitrate range); **M** (Ocean acidification buffering potential); **N** (pH sensitivity); **O** (Phosphate range); **P** (pO2 tolerance); **Q** (Reproductive frequency); **R** (Salinity range); **S** (Spatial behavior); **T** (Structural provisioning potential); **U** (Temperature tolerance).

Supplementary Information References

1. Beauchard, O., Veríssimo, H., Queirós, A. M. & Herman, P. M. J. The use of multiple biological traits in marine community ecology and its potential in ecological indicator development. Ecological Indicators 76, 81–96 (2017).
2. Grebe, G. S. Evaluating and Advancing Opportunities for Ecosystem Services, Increased Production, and Sustainability of Kelp Aquaculture in the Gulf of Maine. (The University of Maine, 2021).
3. Bartley, D. M. World Aquaculture 2020–A brief overview. (2022).
4. Clarke, R. & Bostock, J. Regional Review on Status and Trends in Aquaculture Development in Europe – 2015. (2017).
5. Tacon, A. & Metian, M. Fish Matters: Importance of Aquatic Foods in Human Nutrition and Global Food Supply. Reviews in Fisheries Science 21, (2013).
6. Beveridge, M. C. M. et al. Meeting the food and nutrition needs of the poor: the role of fish and the opportunities and challenges emerging from the rise of aquaculture. Journal of Fish Biology 83, 1067–1084 (2013).
7. Béné, C. et al. Feeding 9 billion by 2050 – Putting fish back on the menu. Food Sec. 7, 261–274 (2015).
8. Craig, R. K. Promoting 'Climate Change Plus' Industries Through the Administrative State: The Case of Marine Aquaculture. SSRN Scholarly Paper at https://doi.org/10.2139/ssrn.3901203 (2022).
9. De Silva, S. S. Regional review on status and trends in aquaculture development in Asia and the Pacific – 2020. (FAO, 2022). doi:10.4060/cb8400en.
10. Romana-Eguia, M. R. R., Parado-Estepa, F. D., Salayo, N. D. & Lebata-Ramos, M. J. H. Resource enhancement and sustainable aquaculture practices in Southeast Asia: challenges in responsible production of aquatic species : proceedings of the international workshop on resource enhancement and sustainable aquaculture practices in Southeast Asia 2014 (RESA). (Aquaculture Department, Southeast Asian Fisheries Development Center, 2015).
11. Pham, T. T. T. et al. Guidelines for co-creating climate adaptation plans for fisheries and aquaculture. Climatic Change 164, 62 (2021).
12. Diana, J. S. Aquaculture Production and Biodiversity Conservation. BioScience 59, 27–38 (2009).
13. Gjedrem, T. Genetic improvement for the development of efficient global aquaculture: A personal opinion review. Aquaculture 344–349, 12–22 (2012).
14. FAO Fisheries and Aquaculture Department. The state of world fisheries and aquaculture 2022: towards blue transformation. (Food and Agriculture Organization of the United Nations, 2022). https://doi.org/10.4060/cc0461en
15. Kelleher, K. Aquaculture: Changing the Face of the Waters, Meeting the Promise and Challenge of Sustainable Aquaculture. (2007). doi:10.1596/978-0-8213-7015-5.

16. Clarke, T. M. et al. Aerobic growth index (AGI): An index to understand the impacts of ocean warming and deoxygenation on global marine fisheries resources. Progress in Oceanography 195, 102588 (2021).
17. Morée, A. L., Clarke, T. M., Cheung, W. W. L. & Frölicher, T. L. Impact of deoxygenation and warming on global marine species in the 21st century. Biogeosciences 20, 2425–2454 (2023).
18. Cheung, W., Lam, V. & Pauly, D. Dynamic bioclimate envelope model to predict climate-induced changes in distribution of marine fishes and invertebrates. Modelling Present and Climate-shifted Distributions of Marine Fishes and Invertebrates 16, 5–50 (2008).
19. Pitcher, T. J. Fish Schooling. in Encyclopedia of Ocean Sciences (ed. Steele, J. H.) 975–987 (Academic Press, 2001). doi:10.1006/rwos.2001.0022.
20. Maire, E. et al. Micronutrient supply from global marine fisheries under climate change and overfishing. Current Biology 31, 4132-4138.e3 (2021).
21. Beal, T., Massiot, E., Arsenault, J. E., Smith, M. R. & Hijmans, R. J. Global trends in dietary micronutrient supplies and estimated prevalence of inadequate intakes. PLoS One 12, e0175554 (2017).
22. Black, R. E. et al. Maternal and child undernutrition and overweight in low-income and middle-income countries. Lancet 382, 427–451 (2013).
23. Whiting, S. J., Calvo, M. S. & Vatanparast, H. Chapter 43 - Current Understanding of Vitamin D Metabolism, Nutritional Status, and Role in Disease Prevention. in Nutrition in the Prevention and Treatment of Disease (Fourth Edition) (eds. Coulston, A. M., Boushey, C. J., Ferruzzi, M. G. & Delahanty, L. M.) 937–967 (Academic Press, 2017).
24. *Dietary Reference Intakes: The Essential Guide to Nutrient Requirements*. (National Academies Press, 2006). doi:10.17226/11537.
25. U.S. Food and Drug Administration. The lows and highs of percent daily value on the new nutrition facts label. (USFDA, 2022). https://www.fda.gov/food/new-nutrition-facts-label/lows-and-highs-percent-daily-value-new-nutrition-facts-label#:~:text=As%20a%20general%20guide%3A,per%20serving%20is%20considered%20high .
26. Hurrell, R. & Egli, I. Iron bioavailability and dietary reference values. Am J Clin Nutr 91, 1461S-1467S (2010).
27. Pereira, L. A review of the nutrient composition of selected edible seaweeds. in Seaweed: Ecology, Nutrient Composition and Medicinal Uses 15–47 (2011).
28. Peñalver, R. et al. Seaweeds as a Functional Ingredient for a Healthy Diet. Mar Drugs 18, 301 (2020).
29. MacArtain, P., Gill, C. I. R., Brooks, M., Campbell, R. & Rowland, I. R. Nutritional value of edible seaweeds. Nutr Rev 65, 535–543 (2007).

30. Kroeker, K. J. et al. Impacts of ocean acidification on marine organisms: quantifying sensitivities and interaction with warming. Global Change Biology 19, 1884–1896 (2013).
31. Wittmann, A. & Pörtner, H.-O. Sensitivities of extant animal taxa to ocean acidification. Nature Climate Change 3, 995–1001 (2013).
32. Cheung, W. W. L., Pitcher, T. J. & Pauly, D. A fuzzy logic expert system to estimate intrinsic extinction vulnerabilities of marine fishes to fishing. Biological Conservation 124, 97–111 (2005).
33. Romano, N. & Zeng, C. Acute toxicity of ammonia and its effects on the haemolymph osmolality, ammonia-N, pH and ionic composition of early juvenile mud crabs, Scylla serrata (Forskål). *Comparative Biochemistry and Physiology Part A: Molecular & Integrative Physiology* **148**, 278–285 (2007).
34. Maulu, S. et al. Climate Change Effects on Aquaculture Production: Sustainability Implications, Mitigation, and Adaptations. Frontiers in Sustainable Food Systems 5, (2021).
35. Henriksson, P. J. G. et al. Interventions for improving the productivity and environmental performance of global aquaculture for future food security. One Earth 4, 1220–1232 (2021).
36. Finfgeld-Connett D (2010) Generalizability and transferability of meta-synthesis research findings. Journal of Advanced Nursing 66(2): 246–254.
37. Dixon-Woods, M. *et al.* Conducting a critical interpretive synthesis of the literature on access to healthcare by vulnerable groups. *BMC Medical Research Methodology* **6**, 35 (2006).
38. King, G., Keohane, R. & Verba, S. The Importance of Research Design in Political Science. American Political Science Review 89, 454–481 (1995).
39. Finfgeld-Connett D, Johnson ED (2013) Literature search strategies for conducting knowledge-building and theory-generating qualitative systematic reviews. Journal of Advanced Nursing 69(1): 194–204.
40. Finfgeld-Connett, D. Use of content analysis to conduct knowledge-building and theory-generating qualitative systematic reviews. *Qualitative Research* **14**, 341–352 (2014).
41. Joint FAO/WHO/UNU Expert Consultation on Protein and Amino Acid Requirements in Human Nutrition (2002 : Geneva, S., Nations, F. and A. O. of the U., Organization, W. H. & University, U. N. Protein and amino acid requirements in human nutrition : report of a joint FAO/WHO/UNU expert consultation. https://apps.who.int/iris/handle/10665/43411 (2007).
